# Association of technologically assisted integrated care with clinical outcomes in type 2 diabetes in Hong Kong using the prospective JADE Program: A retrospective cohort analysis

**DOI:** 10.1371/journal.pmed.1003367

**Published:** 2020-10-02

**Authors:** Lee-Ling Lim, Eric S. H. Lau, Risa Ozaki, Harriet Chung, Amy W. C. Fu, Wendy Chan, Alice P. S. Kong, Ronald C. W. Ma, Wing-Yee So, Elaine Chow, Kitty K. T. Cheung, Tiffany Yau, C. C. Chow, Vanessa Lau, Rebecca Yue, Shek Ng, Benny Zee, William Goggins, Brian Oldenburg, Philip M. Clarke, Maggie Lau, Rebecca Wong, C. C. Tsang, Edward W. Gregg, Hongjiang Wu, Peter C. Y. Tong, Gary T. C. Ko, Andrea O. Y. Luk, Juliana C. N. Chan

**Affiliations:** 1 Department of Medicine and Therapeutics, The Chinese University of Hong Kong, Prince of Wales Hospital, Shatin, Hong Kong SAR, China; 2 Asia Diabetes Foundation, Shatin, Hong Kong SAR, China; 3 Department of Medicine, Faculty of Medicine, University of Malaya, Kuala Lumpur, Malaysia; 4 Hong Kong Institute of Diabetes and Obesity, The Chinese University of Hong Kong, Prince of Wales Hospital, Shatin, Hong Kong SAR, China; 5 Li Ka Shing Institute of Health Sciences, The Chinese University of Hong Kong, Prince of Wales Hospital, Shatin, Hong Kong SAR, China; 6 Hospital Authority Head Office, Hong Kong SAR, China; 7 Jockey Club School of Public Health and Primary Care, The Chinese University of Hong Kong, Prince of Wales Hospital, Shatin, Hong Kong SAR, China; 8 Centre for Health Policy, Melbourne School of Population and Global Health, The University of Melbourne, Melbourne, Victoria, Australia; 9 Health Economics Research Centre, Nuffield Department of Population Health, University of Oxford, Oxford, United Kingdom; 10 Alice Ho Miu Ling Nethersole Hospital, Tai Po, Hong Kong SAR, China; 11 School of Public Health, Imperial College London, London, United Kingdom; John Hunter Hospital, AUSTRALIA

## Abstract

**Background:**

Diabetes outcomes are influenced by host factors, settings, and care processes. We examined the association of data-driven integrated care assisted by information and communications technology (ICT) with clinical outcomes in type 2 diabetes in public and private healthcare settings.

**Methods and findings:**

The web-based Joint Asia Diabetes Evaluation (JADE) platform provides a protocol to guide data collection for issuing a personalized JADE report including risk categories (1–4, low–high), 5-year probabilities of cardiovascular-renal events, and trends and targets of 4 risk factors with tailored decision support. The JADE program is a prospective cohort study implemented in a naturalistic environment where patients underwent nurse-led structured evaluation (blood/urine/eye/feet) in public and private outpatient clinics and diabetes centers in Hong Kong. We retrospectively analyzed the data of 16,624 Han Chinese patients with type 2 diabetes who were enrolled in 2007–2015. In the public setting, the non-JADE group (*n* = 3,587) underwent structured evaluation for risk factors and complications only, while the JADE (*n* = 9,601) group received a JADE report with group empowerment by nurses. In a community-based, nurse-led, university-affiliated diabetes center (UDC), the JADE-Personalized (JADE-P) group (*n* = 3,436) received a JADE report, personalized empowerment, and annual telephone reminder for reevaluation and engagement. The primary composite outcome was time to the first occurrence of cardiovascular-renal diseases, all-site cancer, and/or death, based on hospitalization data censored on 30 June 2017. During 94,311 person-years of follow-up in 2007–2017, 7,779 primary events occurred. Compared with the JADE group (136.22 cases per 1,000 patient-years [95% CI 132.35–140.18]), the non-JADE group had higher (145.32 [95% CI 138.68–152.20]; *P* = 0.020) while the JADE-P group had lower event rates (70.94 [95% CI 67.12–74.91]; *P* < 0.001). The adjusted hazard ratios (aHRs) for the primary composite outcome were 1.22 (95% CI 1.15–1.30) and 0.70 (95% CI 0.66–0.75), respectively, independent of risk profiles, education levels, drug usage, self-care, and comorbidities at baseline. We reported consistent results in propensity-score–matched analyses and after accounting for loss to follow-up. Potential limitations include its nonrandomized design that precludes causal inference, residual confounding, and participation bias.

**Conclusions:**

ICT-assisted integrated care was associated with a reduction in clinical events, including death in type 2 diabetes in public and private healthcare settings.

## Abstract 2

Lee-Ling Lim and colleagues reveal how personalised medical guidance can reduce clinical outcomes for diabetes patients in Hong Kong.

## Author summary

**Why was this study done?:**

- The public healthcare systems are overburdened with manpower shortage, long waiting time, infrequent structured evaluation, and insufficient patient engagement, while structured patient assessment and education is often not provided in the private sector due to high costs and/or lack of capacity.
- In 2007, we developed the web-based Joint Asia Diabetes Evaluation (JADE) Program, a multicomponent data-driven integrated care program, to close care gaps in both private and public sectors in Hong Kong.
- It combines the use of nonphysician personnel (e.g., nurses), information and communications technology (ICT), and structured evaluation with issue of personalized JADE reports to empower patients and promote shared decision-making.
- In 2007, we also established a community-based, nurse-led diabetes center to complement public and private care in Hong Kong by increasing community access to JADE-assisted evaluation for personalized empowerment and engagement.

**What did the researchers do and find?:**

- This retrospective analysis involved 16,624 patients with type 2 diabetes enrolled from the public and private sector in Hong Kong between 2007 and 2015.
- We categorized these patients into 3 groups, namely non-JADE, JADE, and JADE-Personalized (JADE-P), and compared their risk for clinical events after 6 years of follow-up.
- Compared with the JADE group (publicly funded evaluation with JADE reports and group education), the non-JADE group (publicly funded evaluation only) had 19%–34% higher risk of clinical events, including hospitalization.
- Compared with the JADE group, the JADE-P group (self-paid evaluation with JADE reports, personalized empowerment, and annual telephone reminder for engagement) had 23%–36% lower risk of clinical events, including hospitalization and death.

**What do these findings mean?:**

- Multicomponent, data-driven integrated care, assisted by nonphysician personnel and ICT, is associated with a reduction in clinical events and death in patients with type 2 diabetes.
- Its implementation in the private sector is an affordable option for patients who opt for a more user-friendly and personalized care and may reduce the burden of hospitalization in the public sector.

## Introduction

The silent, progressive, and multisystem nature of diabetes calls for periodic evaluation to avoid delayed intervention [1,2]. Despite advancing knowledge and technologies proven to be efficacious in trial settings, there are huge care gaps in type 2 diabetes in real-world practice [1,3,4]. People with diabetes require life-long follow-up and self-management, which calls for stable patient–provider relationships and ongoing support to sustain behavioral change [1,5]. In low- and middle-income countries/areas, lack of care access is a key challenge [6]. In high-income countries/areas with medical coverage, large patient volume, complex care protocols, frequent changes of healthcare providers (HCPs), lack of regular evaluation, and insufficient patient engagement can lead to delayed intervention, suboptimal self-management, and patient distress with poor clinical outcomes [5,7].

In a meta-analysis of randomized quality improvement programs (QIPs), team-based care, patient education and empowerment, as well as using relay (e.g., nonphysician personnel or technology) to enhance patient–provider communication are most effective in reducing cardiovascular risk factors, especially in developing countries/areas [8], which, if sustained, can be life- and cost-saving [9]. Due to the high patient:HCP ratio, use of information and communications technology (ICT) and nonphysician personnel can improve the efficiency and continuation of care delivery. Since patients have different perspectives, expectations, and values, provision of these QIPs in both public and private healthcare settings may increase their reach and impact [5,10].

In 1995, the Chinese University of Hong Kong (CUHK) initiated a research-driven QIP of using team-based care to establish the Hong Kong Diabetes Register (HKDR) and using the data to stratify risk, triage care, empower patients, and inform decision-making. This data-driven integrated care model has provided the template for a territory-wide risk evaluation and management program, initially at hospital-based diabetes centers in 2000 and later at primary care clinics in 2009 operated by the publicly funded Hospital Authority (HA) [11]. In 2007, the CUHK initiated 2 knowledge transfer projects of using ICT and nonphysician personnel to evaluate, empower, and engage patients for complementing physician care. Firstly, we developed the Joint Asia Diabetes Evaluation (JADE) Technology, which is a web-based platform to guide data collection, followed by issue of a personalized report to facilitate shared decision-making. Secondly, we established a community-based, nurse-led university-affiliated diabetes center (UDC) to offer a self-funded and JADE-Personalized (JADE-P)-assisted program with annual telephone reminder for reevaluation and engagement [11]. In this retrospective analysis of prospectively accrued real-world data, we examined the association of the JADE-P care model with clinical outcomes, along with 2 structured evaluation programs implemented in 2 publicly funded hospitals, one using the JADE Technology (JADE) and the other not using it (non-JADE).

## Methods

### Health system in Hong Kong

Countries/areas have different taxation and health financing policies, although insufficient integration between hospitals and the community, as well as public and private sectors, often lead to care fragmentation. In most countries, the annual growth of healthcare expenditure exceeds that of the growth of gross domestic product (GDP), calling for more efficient and value-added care delivery [12]. Hong Kong has 7.5 million inhabitants, predominantly Han Chinese, with a dual-track healthcare system. The city adopts a low tax system with the salary/corporate tax rate capped at 17%, with 17% of the government revenue capped for healthcare spending, equivalent to 3% GDP [13]. In Hong Kong, the GDP per capita was US$48,675 in 2018 [14], while the median monthly household income (with at least one member being economically active) was US$4,615 in 2019 [15].

The healthcare system in Hong Kong is modelled after the United Kingdom National Health System (UK-NHS), with a single care provider and in that the HA receives annual government funding to operate all publicly funded hospitals and clinics. The HA employs >76,000 staff including 6,000 doctors, which represents half of the medical force but provides >90% of hospital and ambulatory outpatient care [16]. Similar to the UK-NHS (funded by 9.8% of GDP) [17], patients attending HA facilities pay a nominal fee that covers essential medicines, investigations, major procedures, and hospitalizations. In Hong Kong, the total health expenditure has increased from 3.6% of GDP in 1989–1990 to 6.2% in 2017–2018, with the public and private sector sharing half of the total expenditure, i.e., approximately 3% [18]. Compared with the UK-NHS, the lower funding level (3% GDP) means considerable strains on the public healthcare system in Hong Kong [18]. Since medical insurance is not compulsory and many patients with diabetes are denied private medical insurance, most patients requiring long-term care and hospitalizations use the HA services, which has a territory-wide Electronic Medical Record (EMR) system that captures all clinic visits, prescriptions, laboratory tests, and hospitalizations.

### The JADE Program

We have reported the evolution of diabetes care in Hong Kong, driven by research to inform practice and policy [11]. Briefly, in 1995, the CUHK diabetes team initiated a nurse-led structured evaluation for risk factors and complications (including eye, feet, blood, and urine tests) for data collection using a pre-printed form at the Diabetes Center located in the publicly funded Prince of Wales Hospital (PWH), the CUHK teaching hospital [11]. Doctors from all medical clinics referred 30–50 patients weekly for evaluation, and data were used to establish the HKDR. All patients returned in 4–6 weeks to receive a report card with nurse explanation in groups of 20–30 patients, followed by care triage to family doctors/hospital internists. In 2000, the HA adopted the HKDR protocol and reformed the diabetes service by setting up nurse-led diabetes centers in hospitals and training nurses in primary care clinics to provide evaluation, education, and review services that had reached out to 0.8 million patients with diabetes by 2016 [19]. However, only a few centers systematically share the results of the evaluation with the patients [11].

**Fig 1:**
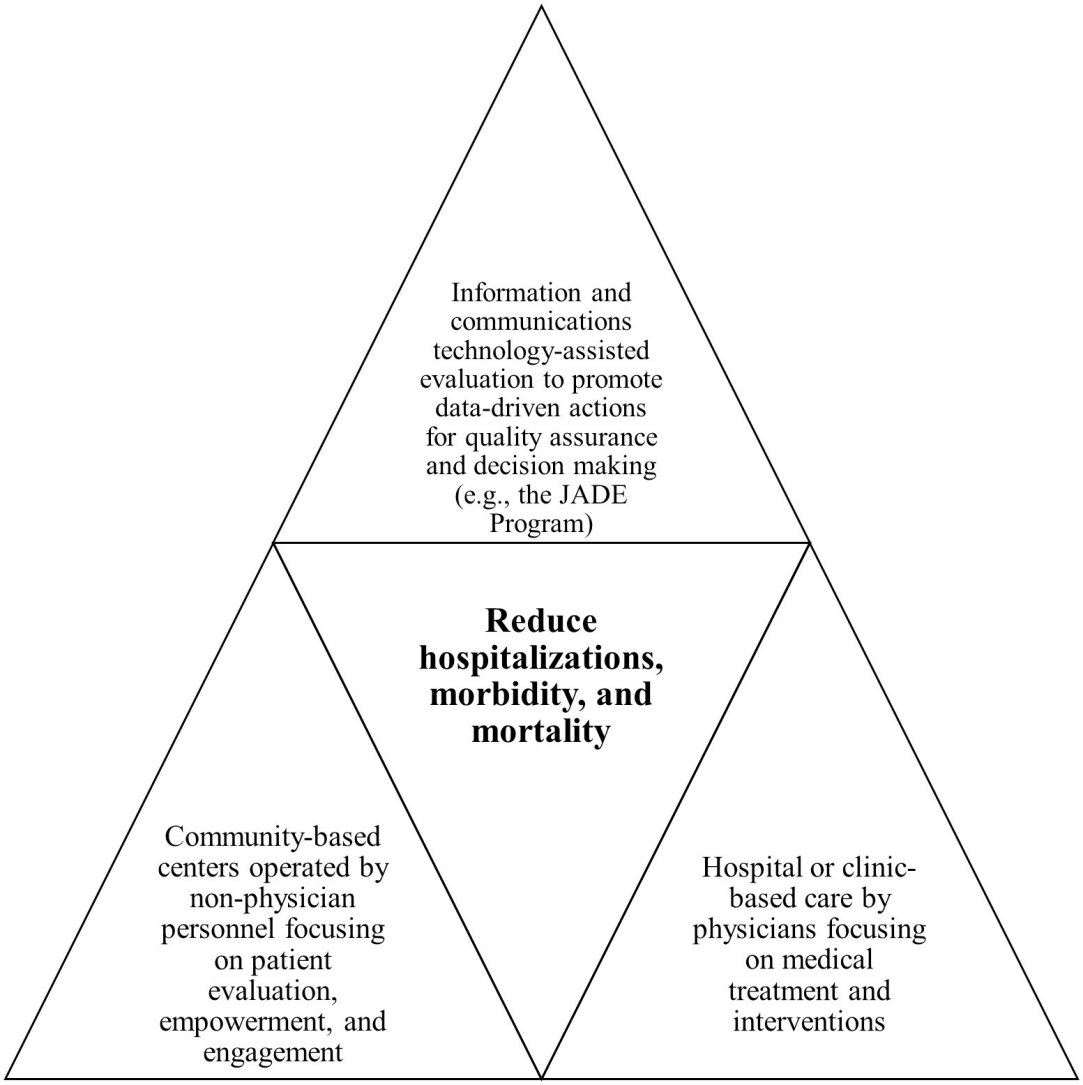
The conceptual framework of the JADE Program. The JADE Program advocates the establishment of hospital- and community-based diabetes centers, as well as the use of ICT and nonphysician personnel to evaluate, empower, and engage patients for providing quality assurance and complementing physician care. ICT, information and communications technology; JADE, Joint Asia Diabetes Evaluation.

The conceptual framework of the JADE Program is to advocate the use of diabetes centers, ICT, and nonphysician personnel to evaluate, empower, and engage patients for providing quality assurance, promoting self-management, and complementing physician care (Fig 1). In 2007, we established the Asia Diabetes Foundation (ADF), a charitable foundation, to develop the web-based JADE Technology, which includes a portal with built-in protocols and HKDR-derived risk algorithms to guide data collection for issue of a personalized JADE report to promote shared decision-making [11]. Pre-printed forms are used to collect data for establishing the JADE Register administered by the ADF. Based on various combinations of risk factors, complications, and risk scores, patients were categorized into risk levels 1 to 4 (low to high risk). The incidence of clinical events (cardiovascular disease [CVD], end-stage renal disease [ESRD], and all-cause death) for risk levels 1 to 4 were 1.99%, 8.17%, 18.54%, and 38.75%, respectively, after a median follow-up of 5.5 years [20]. The JADE report displays the risk categories, future event rates, and trends and targets of 4 modifiable risk factors (blood pressure [BP], hemoglobin A_1c_ [HbA_1c_], low-density lipoprotein [LDL] cholesterol, and body weight) with personalized recommendations for HCPs and patients, focusing on early intervention and self-management, triggered by attained levels (S1 Fig) [11].

### Patients and settings

Using a territory-wide diabetes database, the annual incidence of type 2 diabetes in Hong Kong Chinese was approximately 1% between 2002 and 2015 [21]. In a population-based survey in 2014–2015, the prevalence of diabetes (self-reported physician diagnosed or based on biochemical testing using either fasting plasma glucose ≥7.0 mmol/L or HbA_1c_ ≥ 6.5%) in people aged 15–84 years was 8.4% with 54% being undiagnosed [22]. Public patients rely on their attending doctors for referral to the diabetes centers to undergo evaluation every 2–3 years. A reminder system through EMR was introduced only after 2016. The heavily subsidized public care and insufficient insurance coverage mean many patients with diabetes may see private doctors while receiving chronic medications and acute hospital care from HA. In 2007, supported by philanthropic funds, the CUHK established a community-based, nurse-led UDC to offer a self-funded JADE-assisted evaluation to complement public and private physician care (JADE-P group). Community doctors or patients can refer themselves to the UDC for this self-paid service (approximately US$300), which covers: (1) a 45-minute structured evaluation, (2) a 30-minute individualized nurse explanation of the JADE report with written recommendations by the CUHK endocrinologists, and (3) annual telephone reminder for reevaluation and engagement.

**Fig 2:**
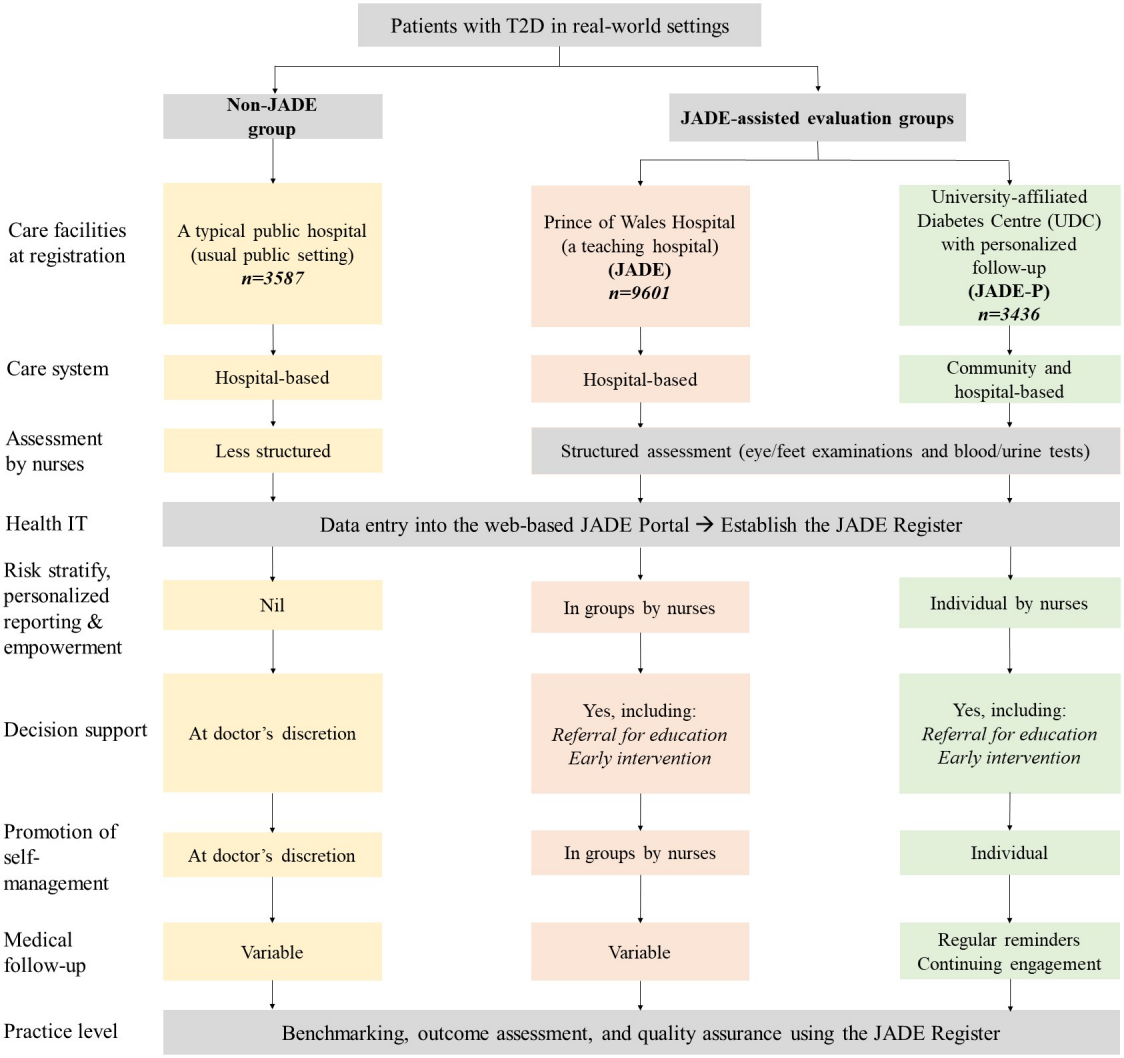
The evaluation workflow of 3 care settings (non-JADE, JADE, and JADE-P). IT, informational technology; JADE, Joint Asia Diabetes Evaluation; JADE-P, JADE-Personalized; T2D, type 2 diabetes.

Since 2007, patients at the PWH, where the HKDR was initiated in 1995, underwent JADE-assisted evaluation every 2–3 years and received the JADE report and group education by nurses (JADE group). After participating in a peer-support project in 2007 [23], nurses in a publicly funded, non-PWH diabetes center entered data into the JADE portal but did not have enough manpower to issue/explain the JADE report (non-JADE group). We compared the clinical outcomes in these 3 settings with different care components summarized as follows: (1) non-JADE: publicly funded evaluation, (2) JADE: publicly funded evaluation, JADE report, and group education, and (3) JADE-P: self-paid evaluation, JADE report, personalized empowerment, and annual telephone reminder for engagement (Fig 2). Patients in all 3 settings used similar evaluation protocol [24]. This is a prospective cohort with documentation of baseline profiles with evaluation of outcomes using the territory-wide EMR in a naturalistic environment. The establishment of the JADE Register with ongoing evaluation was approved by the Joint CUHK-New Territories East Cluster Clinical Research Ethics Committee. All patients provided written informed consent prior to registration into the JADE Program.

### Outcomes

Over the years, using a unique identifier, we periodically retrieved hospitalization, laboratory, and prescription data from the territory-wide EMR system as well as mortality data from the Hong Kong Death Register. The analysis plan employed standard statistical methods in evaluating the associations of predefined risk factors and interventions on clinical outcomes [24,25]. In this retrospective analysis of a prospective cohort registered between 2007 and 2015, we used International Classification of Diseases (ICD)-9-coded hospital discharge data and ICD-10 codes linked to the Hong Kong Death Register to define clinical outcomes (S1 Table) [26]. These hospitalization data from publicly funded hospitals were censored on 30 June 2017 with at least 2 years of observation after enrolment in the JADE Register. The primary composite outcome was time to the first occurrence of any major clinical events, including fatal/nonfatal coronary heart disease (CHD), peripheral vascular disease (PVD), stroke, heart failure, chronic kidney disease (CKD; estimated glomerular filtration rate [eGFR] < 60 mL/min/1.73 m^2^), ESRD (eGFR < 15 mL/min/1.73 m^2^ or need for renal replacement therapy), all-site cancer, and/or all-cause death. We estimated GFR using the creatinine-based CKD-Epidemiological Collaboration equation [27]. The secondary outcomes included the incidence of individual clinical events and all-cause hospitalization. This study is reported as per the Strengthening the Reporting of Observational Studies in Epidemiology (STROBE) guideline (S1 Checklist).

### Statistical analysis

Descriptive data are expressed in mean ± SD, median (IQR), and number (percentage), as appropriate. Triglyceride and urinary albumin:creatinine ratio (ACR) were natural log-transformed. We used χ^2^, Fisher’s exact, one-way ANOVA, or Kruskal-Wallis test for between-group comparisons. We combined JADE risk levels 1 and 2 due to small sample size and reported crude incidence rates as number of cases per 1,000 patient-years with 95% CI. We compared the incidence rates of 3 care settings using the Poisson regression model, with the JADE group as the referent.

We performed multivariable Cox proportional hazards models to obtain the adjusted hazard ratios (aHRs) with 95% CI of the first occurrence of clinical events in the non-JADE and JADE-P versus the JADE group. All Cox models were adjusted for age, sex, diabetes duration, education status, self-care index (self-report of dietary adherence, regular exercise, and self-monitoring of blood glucose), smoking status, HbA_1c_, systolic BP, LDL cholesterol, high-density lipoprotein (HDL) cholesterol, triglyceride, waist circumference, urinary ACR, eGFR, medication usage (oral glucose-lowering drugs, insulin, renin-angiotensin system inhibitors [RASi], and statins), diabetic retinopathy, sensory neuropathy, and comorbidities documented at baseline. Since percutaneous coronary intervention (PCI) might be elective, we excluded patients with PCI and performed a subanalysis including only patients with hospitalization due to myocardial infarction (MI), defined as fatal/nonfatal acute MI or coronary artery bypass graft surgery.

We performed 2 sensitivity analyses. First, we generated 2 sets of propensity scores using confounders that may influence outcomes with 1:1 matching for (1) non-JADE versus JADE and (2) JADE-P versus JADE comparisons (S2 Table). We evaluated the propensity score matching by using standardized differences of variables between the 2 groups post-matching. A standardized difference of less than 0.1 implied negligible difference between the 2 groups (S3 and S4 Tables) [28]. We used Kaplan-Meier analysis with logrank test to estimate survival probabilities for any major clinical event or hospitalization between the propensity-score–matched non-JADE versus JADE, as well as the JADE-P versus JADE groups. Second, patients were categorized as being lost to follow-up when neither laboratory measurement nor hospitalization was captured by the territory-wide EMR system in the public sector 1 year before data censoring or death. We then performed Cox proportional hazards models analysis in patients who were not categorized as lost to follow-up. All analyses were performed using R version 3.3.1 (www.r-project.org) with *P* < 0.05 (2-tailed) as significant.

## Results

### Baseline characteristics

A total of 16,624 patients with type 2 diabetes and 160 endocrinologists, internists, and primary care physicians in the public and private sector participated in the present study. In the entire cohort (age: 60.3 ± 11.6 years, 54.6% men, diabetes duration: 7.0 years [IQR 2.0–14.0]), 78% were managed in the JADE or JADE-P settings. Fewer than 50% were treated with either RASi or statins, and only 30% attained ≥2 treatment targets (HbA_1c_ < 7% [53 mmol/mol], BP < 130/80 mmHg, LDL cholesterol < 2.6 mmol/L) at baseline. Nearly 90% belonged to the high or very high-risk groups (JADE risk levels 3–4). The JADE-P group who attended the UDC were younger and more educated with shorter diabetes duration, fewer complications, and better risk factor control than the JADE and non-JADE groups (Table 1).

**Table 1:** Baseline characteristics of patients with type 2 diabetes categorized by 3 care settings: (1) non-JADE group (publicly funded evaluation), (2) JADE group (publicly funded evaluation with JADE report and group education), and (3) JADE-P group (self-paid evaluation with JADE report, personalized empowerment, and annual telephone reminder for engagement).

	All patients	Non-JADE	JADE	JADE-P
**Number (%)**	16,624 (100%)	3,587 (21.6%)	9,601 (57.8%)	3,436 (20.7%)
Observation period* **(years)**	6.0 (4.2–7.0)	5.5 (3.8–6.7)	5.7 (4.1–6.7)	7.8 (5.7–8.8)
**Age (years)**	60.3 ± 11.6	59.9 ± 11.7	60.9 ± 11.5	58.9 ± 11.4
Diabetes duration* **(years)**	7.0 (2.0–14.0)	7.0 (2.0–14.0)	8.0 (2.0–15.0)	5.0 (1.0–11.0)
**Age of diagnosis (years)**	51.9 ± 11.2	51.3 ± 11.5	52.0 ± 11.3	52.2 ± 10.7
**Men, *n* (%)**	9,071 (54.6%)	1,952 (54.4%)	5,110 (53.2%)	2,009 (58.5%)
**Former/current smoker, *n* (%)**	5,333 (32.1%)	1,258 (35.1%)	3,014 (31.4%)	1,061 (30.9%)
**At least college education, *n* (%)**	1,899 (11.5%)	288 (8.1%)	934 (9.8%)	677 (19.7%)
**JADE risk level, *n* (%)**				
1–2^†^	1,940 (11.7%)	428 (11.9%)	900 (9.4%)	612 (17.8%)
3	11,012 (66.2%)	2,336 (65.1%)	6,398 (66.7%)	2,278 (66.3%)
4	3,670 (22.1%)	823 (22.9%)	2,301 (24.0%)	546 (15.9%)
**Past medical history, *n* (%)**				
CHD	2,141 (12.9%)	466 (13.0%)	1,287 (13.4%)	388 (11.3%)
PVD	641 (3.9%)	96 (2.7%)	455 (4.7%)	90 (2.6%)
Stroke	1,421 (8.6%)	349 (9.7%)	877 (9.1%)	195 (5.7%)
Heart failure	613 (3.7%)	110 (3.1%)	439 (4.6%)	63 (1.8%)
CKD	3,212 (19.3%)	716 (20.0%)	2,023 (21.1%)	473 (13.8%)
ESRD	265 (1.6%)	80 (2.2%)	171 (1.8%)	14 (0.4%)
Sensory neuropathy	966 (5.8%)	88 (2.5%)	722 (7.5%)	156 (4.5%)
Diabetic retinopathy	4,481 (27.0%)	1,078 (30.1%)	2,600 (27.1%)	803 (23.5%)
All-site cancer	994 (6.0%)	187 (5.2%)	639 (6.7%)	168 (4.9%)
**Clinical assessment**				
Waist circumference (men; cm)	91.9 ± 10.8	90.1 ± 10.5	93.0 ± 11.0	90.8 ± 10.0
Waist circumference (women; cm)	87.2 ± 11.2	85.7 ± 11.1	88.2 ± 11.3	85.5 ± 10.5
Systolic BP (mmHg)	136.0 ± 18.8	136.0 ± 18.5	138.0 ± 18.9	131.0 ± 18.3
Diastolic BP (mmHg)	78.2 ± 10.6	76.0 ± 10.4	79.4 ± 10.8	77.3 ± 9.9
**Biochemical assessment**				
HbA_1c_ (%)	7.59 ± 1.58	7.78 ± 1.62	7.60 ± 1.55	7.37 ± 1.60
HbA_1c_ (mmol/mol)	59.0 ± 17.3	62.0 ± 17.7	60.0 ± 16.9	57.0 ± 17.5
Triglyceride* (mmol/L)	1.3 (0.9–1.9)	1.4 (1.0–2.0)	1.3 (0.9–1.8)	1.4 (1.0–2.0)
HDL cholesterol (mmol/L)	1.31 ± 0.37	1.34 ± 0.38	1.30 ± 0.38	1.27 ± 0.33
LDL cholesterol (mmol/L)	2.59 ± 0.89	2.77 ± 0.94	2.50 ± 0.84	2.66 ± 0.93
Urinary ACR* (mg/mmol)	1.8 (0.6–8.9)	2.2 (0.7–10.1)	2.1 (0.7–10.9)	1.2 (0.5–4.3)
eGFR (mL/min/1.73 m^2^)	80.1 ± 23.8	81.1 ± 24.9	78.7 ± 24.2	83.2 ± 20.8
**Treatment targets attainment at baseline, *n* (%)**				
HbA_1c_ < 7% (53 mmol/mol)	6,897 (41.6%)	1,310 (36.6%)	3,855 (40.2%)	1,732 (50.4%)
BP < 130/80 mmHg	5,583 (33.6%)	1,310 (36.5%)	2,850 (29.7%)	1,423 (41.4%)
LDL cholesterol < 2.6 mmol/L	8,541 (52.4%)	1,528 (43.3%)	5,357 (57.1%)	1,656 (48.7%)
At least 2 treatment targets attained	6,341 (38.1%)	1,211 (33.8%)	3,558 (37.1%)	1,572 (45.8%)
**Medication usage at baseline, *n* (%)**				
Insulin	4,152 (25.0%)	977 (27.2%)	2,759 (28.7%)	416 (12.1%)
Oral glucose-lowering agents	14,038 (84.4%)	3,049 (85.0%)	8,212 (85.5%)	2,777 (80.8%)
RASi	7,739 (46.6%)	1,468 (40.9%)	4,949 (51.5%)	1,322 (38.5%)
Statins	7,484 (45.0%)	1,303 (36.3%)	5,011 (52.2%)	1,170 (34.1%)
**Self-care activity in last 3 months, *n* (%)**				
SMBG at least weekly	7,986 (52.0%)	1,560 (48.2%)	4,786 (53.5%)	1,640 (51.5%)
Physical activity at least 3 times/week	7,792 (47.0%)	1,294 (36.6%)	4,884 (50.9%)	1,614 (47.0%)
Adherence to balanced diet	14,905 (89.9%)	3,177 (88.8%)	8,752 (91.4%)	2,976 (86.6%)
At least 2 self-care activities	11,050 (66.5%)	2,070 (57.7%)	6,743 (70.2%)	2,237 (65.1%)

*Data are expressed in mean ± SD, median (IQR), and number (percentages), as appropriate.

^†^We combined JADE risk levels 1 and 2 due to small sample size. SI conversion factors: To convert LDL cholesterol and HDL cholesterol to mg/dL, multiply by 38.67. To convert triglyceride to mg/dL, multiply by 88.57.

**Abbreviations**: ACR, albumin:creatinine ratio; BP, blood pressure; CHD, coronary heart disease; CKD, chronic kidney disease; DPP4, dipeptidyl peptidase-4; eGFR, estimated glomerular filtration rate; ESRD, end-stage renal disease; HbA_1c_, hemoglobin A_1c_; HDL, high-density lipoprotein; JADE, Joint Asia Diabetes Evaluation; JADE-P, JADE-Personalized; LDL, low-density lipoprotein; NA, not applicable; PVD, peripheral vascular disease; RAS, renin-angiotensin system; SI, International System of Units; SMBG, self-monitoring of blood glucose

### Outcomes

After a median follow-up of 6.0 years (IQR 4.2–7.0; 94,311 person-years), 7,779 patients developed at least an incident event. There were 1,523 deaths and 6,960 CKD, 1,051 CHD, 641 heart-failure, and 623 stroke events (Table 2). Before adjusting for differences in baseline factors, the non-JADE group had higher event rates, especially CKD and hospitalization, than the JADE group. The JADE-P group had lower rates of all major clinical events, including all-cause death and hospitalization, than the JADE group. Similar differences were observed when stratified by JADE risk levels (S5 Table). For cause-specific deaths, the JADE-P group had lower incidence of vascular, cancer, and nonvascular and noncancer deaths than the JADE group (S6 Table). The distribution of cause-specific deaths between the non-JADE and JADE groups were similar.

**Table 2:** Incidence rates of clinical events and hospitalization (cases per 1,000 patient-years) in all patients with type 2 diabetes managed in 3 care settings, namely non-JADE, JADE-P, and JADE (referent).

	All patients	JADE (publicly funded evaluation, JADE report, and group education; referent group)	Non-JADE (publicly funded evaluation)	*P* value (non-JADE versus JADE)	JADE-P (self-paid evaluation, JADE report, personalized empowerment, and annual telephone reminder for engagement)	*P* value (JADE-P versus JADE)
**Any major clinical events**						
Events (*n*)	7,779	4,692	1,796	N/A	1,291	N/A
Incidence rate	119.67 (117.03–122.36)	136.22 (132.35–140.18)	145.32 (138.68–152.20)	0.020	70.94 (67.12–74.91)	<0.001
**All-cause death**						
Events (*n*)	1,523	947	329	N/A	247	N/A
Incidence rate	16.15 (15.35–16.98)	18.36 (17.21–19.57)	17.81 (15.94–19.84)	0.631	10.18 (8.95–11.53)	<0.001
**CHD**						
Events (*n*)	1,051	620	212	N/A	219	N/A
Incidence rate	11.52 (10.83–12.24)	12.43 (11.47–13.45)	11.80 (10.27–13.50)	0.515	9.36 (8.16–10.68)	<0.001
**MI**						
Events (*n*)	445	273	109	N/A	63	N/A
Incidence rate	4.77 (4.34–5.23)	5.35 (4.74–6.02)	5.97 (4.90–7.20)	0.335	2.62 (2.01–3.35)	<0.001
**PVD**						
Events (*n*)	303	183	73	N/A	47	N/A
Incidence rate	3.24 (2.88–3.62)	3.58 (3.08–4.14)	3.98 (3.12–5.01)	0.437	1.95 (1.43–2.59)	<0.001
**Stroke**						
Events (*n*)	623	350	144	N/A	129	N/A
Incidence rate	6.72 (6.20–7.27)	6.90 (6.19–7.66)	7.94 (6.70–9.35)	0.155	5.41 (4.52–6.43)	0.018
**Heart failure**						
Events (*n*)	641	406	140	N/A	95	N/A
Incidence rate	6.90 (6.38–7.46)	8.01 (7.25–8.83)	7.69 (6.47–9.07)	0.673	3.96 (3.20–4.84)	<0.001
**CKD**						
Events (*n*)	6,960	4,228	1,623	N/A	1,109	N/A
Incidence rate	102.84 (100.44–105.28)	117.73 (114.21–121.34)	126.37 (120.29–132.67)	0.015	58.60 (55.20–62.15)	<0.001
**ESRD**						
Events (*n*)	1,451	893	351	N/A	207	N/A
Incidence rate	15.90 (15.09–16.74)	17.96 (16.80–19.18)	19.80 (17.78–21.98)	0.122	8.69 (7.55–9.96)	<0.001
**All-site cancer**						
Events (*n*)	896	537	176	N/A	183	N/A
Incidence rate	9.69 (9.06–10.34)	10.63 (9.75–11.56)	9.68 (8.30–11.22)	0.283	7.69 (6.62–8.89)	<0.001
**Hospitalization**						
Events (*n*)	8,600	5,093	2,014	N/A	1,493	N/A
Incidence rate	131.71 (128.94–134.52)	144.89 (140.93–148.92)	166.30 (159.12–173.73)	<0.001	82.79 (78.64–87.10)	<0.001

Any major clinical event was defined as the first occurrence of either CHD, PVD, stroke, heart failure, CKD, ESRD, all-site cancer, or all-cause death. MI was defined as either acute MI or coronary artery bypass graft surgery. Hospitalization was calculated for patients with at least an overnight stay. We compared the incidence rates of clinical events and hospitalization using the Poisson regression model, with the JADE group as the referent.

**Abbreviations**: CHD, coronary heart disease; CKD, chronic kidney disease; ESRD, end-stage renal disease; JADE, Joint Asia Diabetes Evaluation; JADE-P, JADE-Personalized; MI, myocardial infarction; N/A, not applicable; PVD, peripheral vascular disease

**Fig 3:**
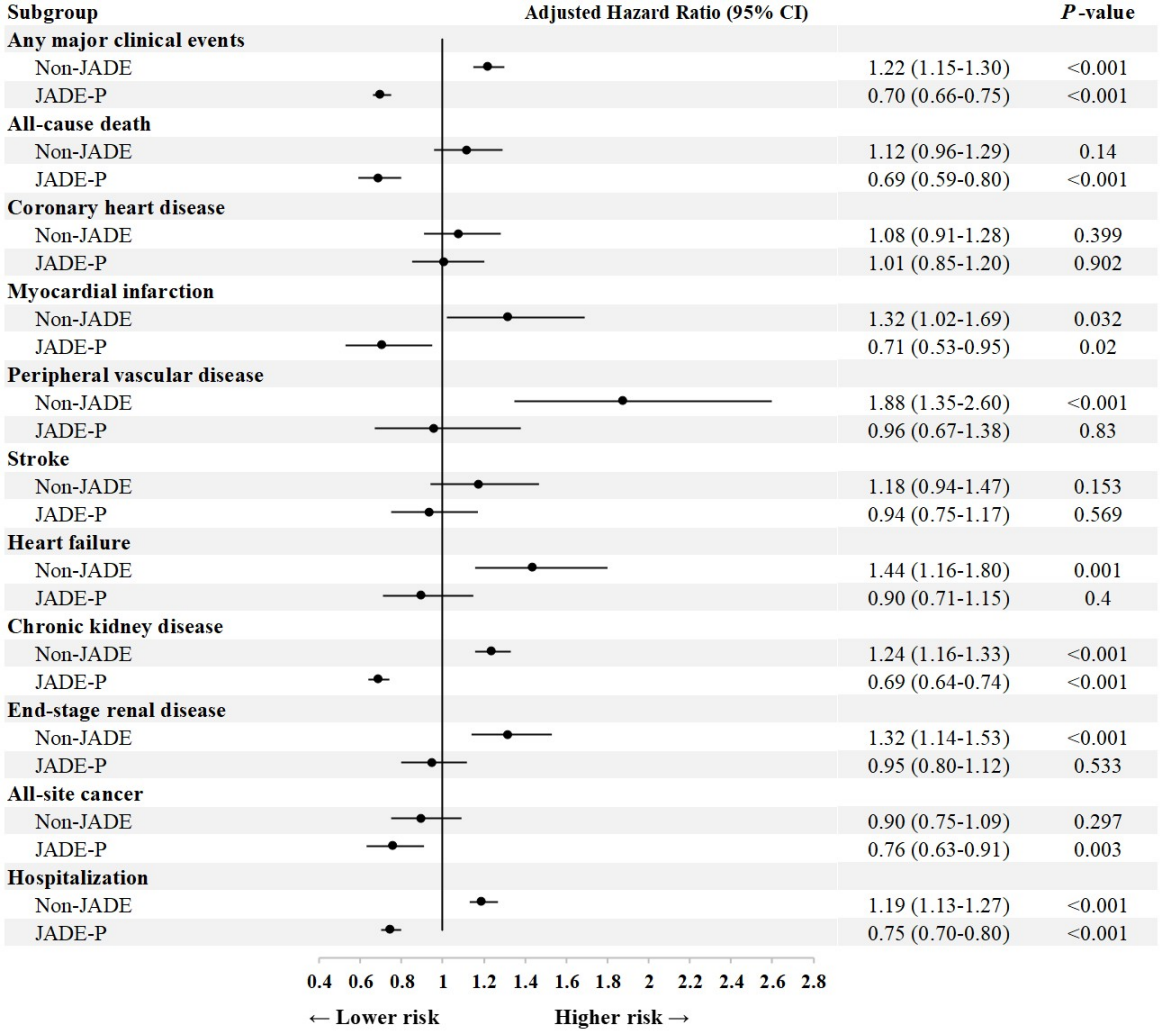
Forest plot showing the independent risk associations of incident clinical events and hospitalization in the non-JADE and JADE-P groups, compared with the JADE group. The non-JADE group underwent publicly funded evaluation. The JADE group received publicly funded evaluation with JADE report and group education. The JADE-P group received self-paid evaluation with JADE report, personalized empowerment, and annual telephone reminder for engagement. Any major clinical event was defined as the first occurrence of either CHD, PVD, stroke, heart failure, CKD, ESRD, all-site cancer, or all-cause death. MI was defined as either acute MI or coronary artery bypass graft surgery. Hospitalization was calculated for patients with at least an overnight stay. We performed multivariable Cox proportional hazards models, adjusting for age, sex, diabetes duration, education status, self-care index, smoking status, HbA_1c_, systolic BP, LDL cholesterol, HDL cholesterol, triglyceride, waist circumference, urinary ACR, eGFR, medication usage (oral glucose-lowering agents, insulin, RASi, and statins), diabetic retinopathy, sensory neuropathy, and comorbidities documented at baseline. Self-care index was defined as any 2 of the following 3 self-care activities, namely self-monitoring of blood glucose at least weekly, adherence to balanced diet, and physical activity at least thrice weekly. ACR, albumin:creatinine ratio; BP, blood pressure; CHD, coronary heart disease; CKD, chronic kidney disease; eGFR, estimated glomerular filtration rate; ESRD, end-stage renal disease; HbA_1c_, hemoglobin A_1c_; HDL, high-density lipoprotein; JADE, Joint Asia Diabetes Evaluation; JADE-P, JADE-Personalized; LDL, low-density lipoprotein; MI, myocardial infarction; PVD, peripheral vascular disease; RASi, renin-angiotensin system inhibitors.

In a multivariable Cox model, the JADE-P group (aHR 0.70, 95% CI 0.66–0.75) had lower risk of primary composite outcome than the JADE group, including all-cause death (aHR 0.69, 95% CI 0.59–0.80), CKD (aHR 0.69, 95% CI 0.64–0.74), all-site cancer (aHR 0.76, 95% CI 0.63–0.91), and hospitalization (aHR 0.75, 95% CI 0.70–0.80). The non-JADE group had higher risk of primary composite outcome (aHR 1.22, 95% CI 1.15–1.30) than the JADE group, including heart failure (aHR 1.44, 95% CI 1.16–1.80), CKD (aHR 1.24, 95% CI 1.16–1.33), ESRD (aHR 1.32, 95% CI 1.14–1.53), and hospitalization (aHR 1.19, 95% CI 1.13–1.27) (Fig 3).

Propensity-score–matched analyses for non-JADE versus JADE (S2 Fig) and JADE-P versus JADE (S3 Fig) yielded similar results. A total of 7.6% patients were considered lost to follow-up. They were more likely to be in the JADE-P group and had lower risk profile than those with available data in the HA EMR system (S7 Table). We also reported similar results after accounting for loss to follow-up and their baseline differences (S4 Fig). At study end, compared with the JADE group, the non-JADE group (S8 Table) had worse, while the JADE-P group had similar, values of risk factors (S9 Table).

**Fig 4:**
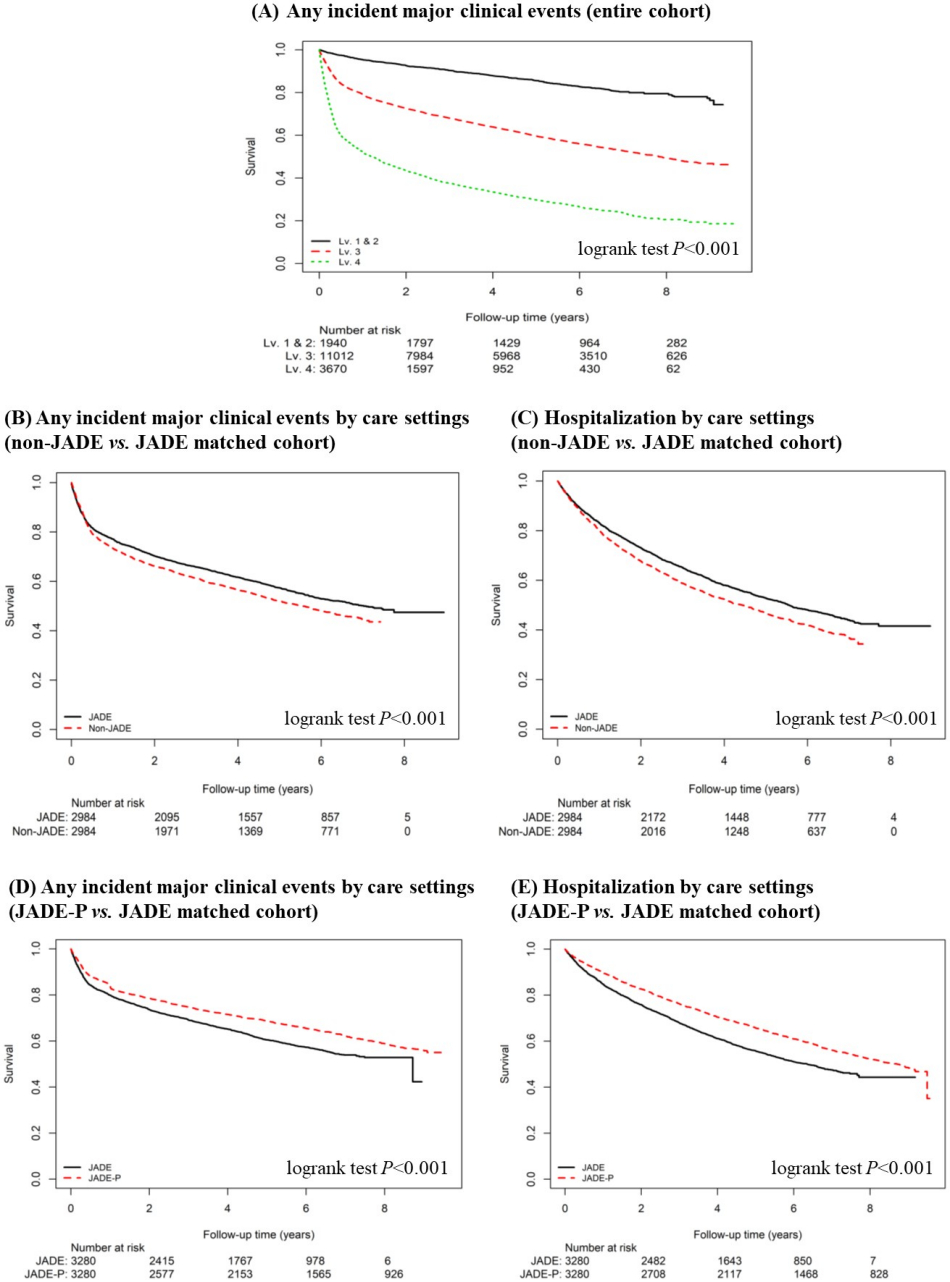
Kaplan-Meier plots showing the survival probabilities of the first occurrence of any major clinical events in the entire cohort, stratified by JADE risk levels (A), and the first occurrence of any major clinical events and hospitalization in the propensity-score–matched cohorts (B–C: non-JADE versus JADE; D–E: JADE-P versus JADE). The non-JADE group underwent publicly funded evaluation. The JADE group received publicly funded evaluation with JADE report and group education. The JADE-P group received self-paid evaluation with JADE report, personalized empowerment, and annual phone reminder for engagement. Any major clinical event was defined as the first occurrence of either CHD, PVD, stroke, heart failure, CKD, ESRD, all-site cancer, or all-cause death. Hospitalization was calculated for patients with at least an overnight stay. Definitions of JADE risk levels [20]: Level 1: No CVD and ESRD; and having other condition fulfilling the “Low-risk” (one or less stratification parameters, and risk scores below the high sensitivity cut-off in all of the risk equations and eGFR ≥ 90 mL/min/1.73 m^2^). Level 2: No CVD and ESRD; none of the conditions (risk score, stratification parameters, eGFR) defined in the “High-risk” category but not belonging to the “Low-risk” category. Level 3: No CVD and ESRD and having 3 or more stratification parameters and/or risk scores above the high specificity cut-off in any one of the risk equations and/or eGFR < 60 mL/min/1.73 m^2^. Level 4: Presence of any CVD (CHD, heart failure, stroke, and/or PVD with or without interventions or medications) and/or ESRD (eGFR < 15 mL/min/1.73 m^2^ or need for renal replacement therapy). CHD, coronary heart disease; CVD, cardiovascular disease; eGFR, estimated glomerular filtration rate; ESRD, end-stage renal disease; JADE, Joint Asia Diabetes Evaluation; JADE-P, JADE-Personalized; Lv, Level; PVD, peripheral vascular disease.

On Kaplan-Meier analysis, the high-risk patients (risk levels 3–4) had higher event rates than the low-risk patients (risk levels 1–2) (Fig 4A). In propensity-score–matched analyses, the non-JADE group had higher rates of any major clinical events and hospitalization than the matched JADE group (Fig 4B and 4C), while both rates were lower in the JADE-P than the matched JADE group (Fig 4D and 4E). In economic evaluation, compared with the JADE care setting, the non-JADE setting would incur an additional cost at US$254 per patient per year, while the JADE-P care setting would save US$880 per patient per year (S1 Text).

## Discussion

In this retrospective analysis of a prospective cohort study conducted in a naturalistic environment, all patients underwent structured evaluation by nurses using the same protocol implemented in different settings. Using the JADE group (publicly funded evaluation, JADE report, and group education) as the referent, the non-JADE group (publicly funded evaluation only) had 19%–34% higher risk for any major clinical events and hospitalization. By contrast, the JADE-P (self-paid evaluation, JADE report, personalized empowerment, and annual telephone reminder for engagement) had 23%–36% lower risk of clinical events, including death, CKD, all-site cancer, and hospitalization, compared with the JADE group. The benefits of JADE and JADE-P care models remained significant on multivariable Cox regression and propensity-score–matched analyses, as well as after excluding patients without data in the public sector who had lower risk profile at baseline. These results accord with the known benefits of using multicomponent strategies to improve patient–provider communication and patient engagement [8]. Using the Reach, Effectiveness, Adoption, Implementation, and Maintenance (RE-AIM) framework, the JADE Program provides a prototype in which the efficient use of ICT and nonphysician personnel, focusing on using data to empower self-management and personalize treatment, can close care gaps with positive outcomes in both public and private healthcare settings [5,29,30].

The silent nature of diabetes with its phenotypic heterogeneity and complexity of management calls for periodic evaluation to enable timely intervention from an interdisciplinary team, including but not limited to specialists, family doctors, healthcare assistants, community health workers, and trained peers [2,31]. Despite increasing investment in outpatient care [32], delayed intervention, lack of ongoing support for health behavior change, and treatment nonadherence due to insufficient engagement and reinforcement mean missed opportunities for early intervention [7,33,34]. In a meta-analysis, 23.6 hours of contact with HCPs is required to sustain 1% reduction in HbA_1c_ over 1 year [35].

The JADE Program and the community-based, nurse-led UDC are academia-led QIPs supported by philanthropic and research funds, which focus on structured evaluation, patient empowerment, and continuing engagement. Our results show that using the JADE Technology to guide data collection for issuing personalized reports is associated with reduced event and death rates, which can complement physician care in the private sector and reduce hospitalizations in the public sector. In Italy, an annual visit to a diabetes center was associated with lower death rates than care by family doctors alone [36]. By using the community-based, nurse-led UDC to provide yearly JADE-assisted evaluation program with flexible scheduling, individualized education and empowerment, and regular reminders, patients who prefer a user-friendly and personalized service may have an affordable option. Although the JADE-P group had better risk profiles and lower usage of medications than the non-JADE and JADE-P groups, these differences were only modest. After adjusting for these differences, the JADE-P group had the best clinical outcomes. Since some patients in the JADE-P group may be using private service, without access to their medical records, we might have missed major events treated in the private sector. However, given the heavily subsidized nature of the public sector, the majority of patients used public service for major events [16]. Besides, the Hong Kong Death Register captures all deaths occurring in Hong Kong. In the sensitivity analysis, we excluded patients considered to be lost to follow-up due to lack of data in the public sector, and the benefits of the JADE-P model remained robust. Taken together, these data suggested that the JADE-P model can add value by reducing all-cause death and hospitalization burden in the public sector while providing an affordable service to complement physician care in both public and private sectors. In this light, frequent changes of HCPs are known to be associated with higher hospitalization and death rates compared with continuing care using nurses and ICT [37,38].

Despite the efficacy of many interventions for diabetes in trial settings, this evidence is not translated fast and efficiently enough to real-world practice [1,5]. Quality aside, affordability and sustainability are important considerations. For chronic and silent diseases such as diabetes, patients often cannot afford or are not willing to pay for expensive private care despite its convenience and personalized service [39]. While high-income and low- and middle-income countries/areas face different challenges in healthcare delivery, improving patient–provider communication, care continuity, patient adherence, and quality assurance are core elements [1,8,30]. The United Nations and World Health Organization advocate adequate coverage to ensure equitable access to healthcare, patient education, and medications, although effective implementation is challenging [30,40]. In low- and middle-income countries/areas, insufficient investment in preventive care, lack of capacity and an interconnected information system, and health illiteracy have contributed to poor control of risk factors and systems performance with high default rates [3,41]. In high-income countries/areas, despite medical coverage and data linkage, high patient:HCP ratio calls for workflow re-engineering and use of nonphysician personnel and technology with strong feedback loops to improve efficiency and quality [5,11,30]. That said, institutional support and reward systems are needed for adoption and sustainability [5,30].

The JADE Technology is designed to translate data-driven integrated care to practice. By enabling nonphysician personnel to evaluate, empower, and engage patients, we can bring out the best of physician care [11]. In a series of QIPs and pragmatic randomized trials, we have reported the benefits of the JADE Technology in improving health literacy, self-management, treatment adherence, negative emotions, risk factor control, and prescription of organ-protective medications [23,41,42]. Setting up diabetes registers such as the JADE Register can identify care gaps, prioritize interventions, benchmark performance, and promote collaborative research [11]. These registers can be linked to hospitalization data and death registers to monitor disease trends and inform planning of healthcare services [10,11,43]. Our data add weight to the growing body of evidence regarding the potential long-term benefits of ICT-assisted integrated care on reducing morbidity and premature death.

To implement these multicomponent integrated care programs, the settings and teams are important considerations. In this analysis, we have demonstrated how we initiated research-driven QIPs, including training of nonphysician personnel, and used data to drive actions for continuous improvement. Apart from the value-added nature of the JADE Program in the public setting, patients who opted for the JADE-P care model in the community-based, nurse-led UDC experienced the best outcomes. In Hong Kong, these research-driven QIPs have motivated the reform of diabetes service in the public setting and provided a prototype for the government to commission nongovernmental organizations to operate community-based centers, run by nonphysician personnel including nurses [44]. These centers provide risk evaluation, education, and supporting programs for diabetes and chronic diseases aimed at complementing public and private physician care [44]. Using the territory-wide EMR, we have recently reported 50%–75% decline in all-cause death and diabetes-related complications in nearly 0.8 million people with diabetes in Hong Kong in 2001–2016 [19,45]. However, the observed decline was less in the 20–44 age group who are known to have worse risk factor control and a higher default rate than their older peers [19,45]. Worryingly, while the incidence of diabetes has declined or plateaued in the middle and older age groups, the incidence of diabetes was increasing in those under the age of 40 [21]. Whether a more user-friendly, personalized, and affordable service such as the JADE-P care model may provide an alternative solution to people of working age requires further exploration.

Our study has several limitations. First, this is a retrospective analysis of an ongoing prospective cohort conducted in real-world settings. Thus, the nonrandomized study design precludes inference of causality, although we have matched patients on key variables, including age, sex, duration of diabetes, education levels, and major cardiovascular risk factors. Second, residual confounding is possible due to unmeasured variance, e.g., participation bias, household income levels, and nursing and physicians’ experiences and practices. However, in the sensitivity analyses, the benefits of JADE and JADE-P care models remained significant after adjusting for education levels and self-management. Third, only 12% of our patients belonged to the low-risk group with a small number of events. Longer duration of follow-up is required to evaluate the association of the JADE Program with clinical outcomes in the low-risk group. Fourth, the baseline risk of patients who were considered lost to follow-up in the present analysis was lower than those who continued follow-up, and this could potentially lead to biased estimates of the JADE-P care model. Despite slight attenuation of effects in complete case analysis, results have generally shown consistent benefits of the JADE-P care model. Fifth, we only had access to the HA EMR and might have underestimated the occurrence of events in the private sector. However, due to the huge cost differences, most patients with acute or major events end up being managed in the public setting [4]. Besides, the capture for death rates was complete using the Hong Kong Death Register except for the rare events of death occurring outside Hong Kong. Last, since sight-threatening diabetic retinopathy or macula edema requiring ophthalmologic procedures do not routinely require hospital admission, we might have underestimated these events. Besides, in the public sector, clinical notes of the Department of Ophthalmology are paper-based and not captured in the EMR.

### Conclusion

Value-added care can reduce hospitalizations and save lives [5,10]. Since 1995, we have reported the impacts of team-based care, telephone reminders, peer support, and setting up a register on clinical outcomes in type 2 diabetes [11]. In this analysis, we have demonstrated the sustained benefits of using data to guide continuous improvement implemented through the JADE Program with reduced all-cause death and hospitalizations in the public setting. In the private sector, we also confirmed the feasibility and acceptability of the implementation of technologically assisted, data-driven integrated care to complement physician care.

## Supporting information

S1 Table Definitions of outcomes using the ICD-9-coded hospital discharge data retrieved from the HA EMR and cause-specific deaths using the ICD-10 codes linked to the Hong Kong Death Register. (DOCX)

S2 Table Variables included in the propensity score. (DOCX)

S3 Table Baseline characteristics of patients with type 2 diabetes in the non-JADE and JADE groups (after propensity score matching). (DOCX)

S4 Table Baseline characteristics of patients with type 2 diabetes in the JADE-P and JADE groups (after propensity score matching). (DOCX)

S5 Table Incidence rates of clinical events and hospitalization (cases per 1,000 patient-years) in patients with type 2 diabetes, stratified by care settings and JADE risk levels (before propensity score matching). (DOCX)

S6 Table Cause-specific death rates (cases per 1,000 patient-years) in all patients with type 2 diabetes and stratified by the non-JADE, JADE, and JADE-P groups (before propensity score matching). (DOCX)

S7 Table Baseline characteristics of patients with type 2 diabetes, stratified by the status of loss to follow-up (yes/no) defined as lack of data in the public healthcare system 1 year before censoring or death, and before propensity score matching. (DOCX)

S8 Table The levels of key risk factors at baseline and study end in the non-JADE and JADE groups (after propensity score matching). (DOCX)

S9 Table The levels of key risk factors at baseline and study end in the JADE-P and JADE groups (after propensity score matching). (DOCX)

S1 Fig An example of a simple-to-read JADE personalized report with particular emphasis on treatment to multiple targets to reduce risk of complications by (1) encouraging doctors to use organ-protective medications (e.g., statins and RASi) and referring patients to receive education and (2) empowering patients to improve self-care, perform regular self-monitoring, and adhere to medications. (TIF)

S2 Fig Independent risk associations of incident clinical events and hospitalization in the non-JADE group, compared with the JADE group (before and after propensity score matching). (TIF)

S3 Fig Independent risk associations of incident clinical events and hospitalization in the JADE-P group, compared with the JADE group (before and after propensity score matching). (TIF)

S4 Fig Independent risk associations of incident clinical events and hospitalization in the non-JADE and JADE-P groups, compared with the JADE group, among patients who were not lost to follow-up as defined by availability of data in the public healthcare system. (TIF)

S1 Text Economic evaluation of the present JADE Program. (DOCX)

S1 STROBE Checklist STROBE, Strengthening the Reporting of Observational Studies in Epidemiology. (DOCX)

## Abbreviations

ACR: albumin:creatinine ratio
ADF: Asia Diabetes Foundation
aHR: adjusted hazard ratio
BP: blood pressure
CHD: coronary heart disease
CKD: chronic kidney disease
CUHK: Chinese University of Hong Kong
CVD: cardiovascular disease
eGFR: estimated glomerular filtration rate
EMR: Electronic Medical Record
ESRD: end-stage renal disease
GDP: gross domestic product
HA: Hospital Authority
HbA_1c_: hemoglobin A1c
HCP: healthcare provider
HDL: high-density lipoprotein
HKDR: Hong Kong Diabetes Register
ICD: International Classification of Diseases
ICT: information and communications technology
JADE: Joint Asia Diabetes Evaluation
JADE-P: JADE-Personalized
LDL: low-density lipoprotein
PCI: percutaneous coronary intervention
PVD: peripheral vascular disease
PWH: Prince of Wales Hospital
QIP: quality improvement program
RASi: renin-angiotensin system inhibitors
RE-AIM: Reach, Effectiveness, Adoption, Implementation, and Maintenance
STROBE: Strengthening the Reporting of Observational Studies in Epidemiology
UDC: university-affiliated diabetes center
UK-NHS: United Kingdom National Health System
