# Supplementary material for: Association of technologically assisted integrated care with clinical outcomes in type 2 diabetes in Hong Kong using the prospective JADE Program: A retrospective cohort analysis
Source: PLoS Med. 2020 Oct 2;17(10):e1003367. doi: 10.1371/journal.pmed.1003367 (PMC7531841; doi:10.1371/journal.pmed.1003367)
Supplement: S1 Table — (DOCX) [file pmed.1003367.s001.docx]

**S1 Table.** Definitions of outcomes using the International Classification of Diseases (ICD)-9-coded hospital discharge data retrieved from the Hospital Authority Electronic Medical Record (EMR) and cause-specific deaths using the ICD-10 codes linked to the Hong Kong Death Register.

| **Events** | **Diagnosis** | **ICD code** |
| --- | --- | --- |
| 1. **Hospital discharge data, EMR (ICD-9)** | | |
| Coronary heart disease | Fatal/non-fatal myocardial infarction or ischaemic heart disease | 410-414 |
|  | Need of coronary revascularization or coronary artery bypass surgery | 36 |
|  | Percutaneous transluminal coronary angioplasty or coronary atherectomy | 00.66 |
| Congestive heart failure | Fatal/non-fatal heart failure | 428 |
| Stroke | Ischaemic stroke   - Occlusion and stenosis of precerebral arteries - Occlusion of cerebral arteries - Death due to: transient cerebral ischaemia | 433  434  435 |
|  | Haemorrhagic stroke   - Subarachnoid haemorrhage - Intracerebral haemorrhage - Other and unspecified intracranial haemorrhage | 430  431  432 |
|  | Acute but ill-defined cerebrovascular disease | 436 |
|  | Death due to: other and ill-defined cerebrovascular disease; late effects of cerebrovascular disease | 437-438 |
| Peripheral vascular disease | Peripheral circulatory disorders | 250.7 |
|  | Peripheral vascular disease | 440.2, 440.4, 443.9 |
|  | Gangrene | 785.4 |
|  | Angiopathy in diseases classified elsewhere | 443.81 |
|  | Peripheral vascular disease unspecified | 443.9 |
|  | Peripheral vascular shunt or bypass | 39.29 |
|  | Insertion of non-drug-eluting peripheral vessel stents | 39.90 |
|  | Non-traumatic amputation of lower limb | 84.1 |
| Chronic kidney disease | Estimated glomerular filtration rate <60 ml/min/1.73m^2^ | Clinical measurement |
| End-stage renal disease | Estimated glomerular filtration rate <15 ml/min/1.73m^2^ | Clinical measurement |
|  | Need for dialysis | 39.95 or 54.98 |
|  | Need for renal transplant | 55.6 |
|  | Fatal/non-fatal renal failure | 585, 586 |
| Cancer | Neoplasms | 140-208 |
| 1. **Cause-specific deaths, Hong Kong Death Register (ICD-10)** | | |
| Vascular deaths | G45, I01, I03-I82, I85-I87, I95-I99, F01, Q20-Q28 | |
| Cancer deaths | C00-C97, D00-D09, D37-D48 | |
| Non-vascular, non-cancer deaths | A00-A99, B00-B99, E00-E89, F02-F99, G00-G99, H00-H95, I89, J00-J99, K00-K99, L00-L99, M00-M99, N00-N99, O00-O99, P00-P96, Q00-Q18, Q30-Q99, S00-S99, T00-T88, V00-V99, W00-W99, X00-X99, Y00-Y99, Z00-Z99 | |
| Deaths due to ill-defined causes | R00-R99 | |
