# Supplementary material for: Association of technologically assisted integrated care with clinical outcomes in type 2 diabetes in Hong Kong using the prospective JADE Program: A retrospective cohort analysis
Source: PLoS Med. 2020 Oct 2;17(10):e1003367. doi: 10.1371/journal.pmed.1003367 (PMC7531841; doi:10.1371/journal.pmed.1003367)
Supplement: S2 Table — (DOCX) [file pmed.1003367.s002.docx]

**S2 Table.** Variables included in the propensity score.

| Age |
| --- |
| Sex |
| Duration of diabetes |
| Education status |
| Smoking status |
| HbA_1c_ |
| Systolic blood pressure |
| LDL-cholesterol |
| HDL-cholesterol |
| Triglyceride |
| Waist circumference |
| Urinary albumin:creatinine ratio |
| Estimated glomerular filtration rate |
