# Supplementary material for: Association of technologically assisted integrated care with clinical outcomes in type 2 diabetes in Hong Kong using the prospective JADE Program: A retrospective cohort analysis
Source: PLoS Med. 2020 Oct 2;17(10):e1003367. doi: 10.1371/journal.pmed.1003367 (PMC7531841; doi:10.1371/journal.pmed.1003367)
Supplement: S3 Table — (DOCX) [file pmed.1003367.s003.docx]

**S3 Table.** Baseline characteristics of patients with type 2 diabetes in the non-JADE and JADE groups (after propensity score-matching).

|  | **Non-JADE**  **(n=2984)** | **JADE**  **(n=2984)** | **Standardized**  **difference** |
| --- | --- | --- | --- |
| Age (years) | 60.4±10.4 | 60.2±10.1 | 0.013 |
| Duration of diabetes^*^ (years) | 7.0 (2.0-14.0) | 7.0 (2.0-14.0) | 0.003 |
| Men, n (%) | 1627 (54.5%) | 1627 (54.5%) | <0.001 |
| Former/current smoker, n (%) | 1044 (35.0%) | 1044 (35.0%) | <0.001 |
| At least college education, n (%) | 226 (7.6%) | 226 (7.6%) | <0.001 |
| Waist circumference (men; cm) | 90.0±10.3 | 90.5±10.4 | 0.005 |
| Waist circumference (women; cm) | 85.7±11.1 | 85.1±10.6 | 0.005 |
| Systolic blood pressure (mmHg) | 135.9±18.5 | 136.2±18.7 | 0.013 |
| HbA_1c_ (%) | 7.75±1.57 | 7.75±1.69 | <0.001 |
| HbA_1c_ (mmol/mol) | 61.0±17.2 | 61.0±18.5 | <0.001 |
| Triglyceride^*^ (mmol/L) | 1.4 (1.0-1.9) | 1.3 (0.9-1.9) | 0.035 |
| HDL-cholesterol (mmol/L) | 1.35±0.38 | 1.36±0.39 | 0.033 |
| LDL-cholesterol (mmol/L) | 2.78±0.93 | 2.77±0.90 | 0.010 |
| Urinary albumin:creatinine ratio^*^ (mg/mmol) | 2.1 (0.7-9.1) | 1.7 (0.6-7.9) | 0.010 |
| Estimated glomerular filtration rate (ml/min/1.73m^2^) | 81.2±23.3 | 81.1±22.4 | 0.005 |

Footnotes: The non-JADE group underwent publicly-funded evaluation. The JADE group received publicly-funded evaluation with JADE report and group education. Data are expressed in mean±standard deviation, median (interquartile range)^*^, and number (percentages), as appropriate. Estimated glomerular filtration rate was calculated using the Chronic Kidney Disease Epidemiology Collaboration creatinine equation. HDL-cholesterol, high-density lipoprotein cholesterol; JADE, Joint Asia Diabetes Evaluation; LDL-cholesterol, low-density lipoprotein cholesterol; NA, not applicable. SI conversion factors: To convert LDL-cholesterol and HDL-cholesterol to mg/dL, multiply by 38.67. To convert triglyceride to mg/dL, multiply by 88.57.
