# Supplementary material for: Association of technologically assisted integrated care with clinical outcomes in type 2 diabetes in Hong Kong using the prospective JADE Program: A retrospective cohort analysis
Source: PLoS Med. 2020 Oct 2;17(10):e1003367. doi: 10.1371/journal.pmed.1003367 (PMC7531841; doi:10.1371/journal.pmed.1003367)
Supplement: S4 Table — (DOCX) [file pmed.1003367.s004.docx]

**S4 Table.** Baseline characteristics of patients with type 2 diabetes in the JADE-P and JADE groups (after propensity score-matching).

|  | **JADE-P**  **(n=3280)** | **JADE**  **(n=3280)** | **Standardized**  **difference** |
| --- | --- | --- | --- |
| Age (years) | 59.7±10.5 | 59.9±10.3 | 0.019 |
| Duration of diabetes^*^ (years) | 5.0 (1.0-11.0) | 6.0 (2.0-12.0) | 0.026 |
| Men, n (%) | 1929 (58.8%) | 1929 (58.8%) | <0.001 |
| Former/current smoker, n (%) | 1014 (30.9%) | 1014 (30.9%) | <0.001 |
| At least college education, n (%) | 630 (19.2%) | 630 (19.2%) | <0.001 |
| Waist circumference (men; cm) | 90.8±9.9 | 90.8±10.2 | 0.003 |
| Waist circumference (women; cm) | 85.5±10.3 | 85.6±10.4 | 0.003 |
| Systolic blood pressure (mmHg) | 131.9±18.3 | 132.0±16.8 | 0.005 |
| HbA_1c_ (%) | 7.36±1.59 | 7.37±1.43 | 0.009 |
| HbA_1c_ (mmol/mol) | 57.0±17.4 | 57.0±15.6 | 0.009 |
| Triglyceride^*^ (mmol/L) | 1.4 (1.0-1.9) | 1.3 (0.9-1.9) | 0.042 |
| HDL-cholesterol (mmol/L) | 1.27±0.33 | 1.27±0.34 | 0.007 |
| LDL-cholesterol (mmol/L) | 2.66±0.94 | 2.63±0.83 | 0.030 |
| Urinary albumin:creatinine ratio^*^ (mg/mmol) | 1.2 (0.5-4.4) | 1.4 (0.5-5.3) | 0.022 |
| Estimated glomerular filtration rate (ml/min/1.73m^2^) | 82.3±20.3 | 82.1±21 | 0.011 |

Footnotes: The JADE group received publicly-funded evaluation with JADE report and group education. The JADE-P group received self-paid evaluation with JADE report, personalized empowerment, and annual telephone reminder for engagement. Data are expressed in mean±standard deviation, median (interquartile range)^*^, and number (percentages), as appropriate. Estimated glomerular filtration rate was calculated using the Chronic Kidney Disease Epidemiology Collaboration creatinine equation. HDL-cholesterol, high-density lipoprotein cholesterol; JADE, Joint Asia Diabetes Evaluation; LDL-cholesterol, low-density lipoprotein cholesterol; NA, not applicable. SI conversion factors: To convert LDL-cholesterol and HDL-cholesterol to mg/dL, multiply by 38.67. To convert triglyceride to mg/dL, multiply by 88.57.
