# Supplementary material for: Association of technologically assisted integrated care with clinical outcomes in type 2 diabetes in Hong Kong using the prospective JADE Program: A retrospective cohort analysis
Source: PLoS Med. 2020 Oct 2;17(10):e1003367. doi: 10.1371/journal.pmed.1003367 (PMC7531841; doi:10.1371/journal.pmed.1003367)
Supplement: S5 Table — (DOCX) [file pmed.1003367.s005.docx]

**S5 Table.** Incidence rates of clinical events and hospitalization (cases *per* 1000-patient-years) in patients with type 2 diabetes, stratified by care settings and JADE risk levels (before propensity score-matching).

|  | **All patients** | **JADE**  **(publicly-funded evaluation, JADE report, and group education; referent group)** | **Non-JADE**  **(publicly-funded evaluation)** | ***P*-value (non-JADE *vs.* JADE)** | **JADE-P**  **(self-paid evaluation,**  **JADE report, personalized empowerment, and annual telephone reminder for engagement)** | ***P*-value (JADE-P *vs.* JADE)** |
| --- | --- | --- | --- | --- | --- | --- |
| 1. **JADE risk levels 1-2** | | | | | | |
| **Any major clinical events** | | | | | | |
| Events (n) | 336 | 153 | 94 | - | 89 | - |
| Incidence rate | 31.18  (27.94-34.70) | 33.19  (28.14-38.89) | 46.40  (37.50-56.78) | 0.011 | 21.50  (17.27-26.46) | 0.001 |
| **All-cause death** | | | | | | |
| Events (n) | 46 | 25 | 13 | - | 8 | - |
| Incidence rate | 3.89  (2.84-5.18) | 4.96  (3.21-7.33) | 5.66  (3.01-9.68) | 0.700 | 1.78  (0.77-3.50) | 0.011 |
| **Coronary heart disease** | | | | | | |
| Events (n) | 31 | 15 | 4 | - | 12 | - |
| Incidence rate | 2.64  (1.79-3.75) | 3.00  (1.68-4.95) | 1.75  (0.48-4.48) | 0.337 | 2.70  (1.39-4.71) | 0.781 |
| **Myocardial infarction** | | | | | | |
| Events (n) | 7 | 5 | 1 | - | 1 | - |
| Incidence rate | 0.59  (0.24-1.22) | 0.99  (0.32-2.32) | 0.44  (0.01-2.43) | 0.451 | 0.22  (0.01-1.24) | 0.171 |
| **Stroke** | | | | | | |
| Events (n) | 22 | 10 | 5 | - | 7 | - |
| Incidence rate | 1.87  (1.17-2.82) | 1.99  (0.96-3.66) | 2.19  (0.71-5.10) | 0.866 | 1.56  (0.63-3.22) | 0.620 |
| **Heart failure** | | | | | | |
| Events (n) | 7 | 2 | 1 | - | 4 | - |
| Incidence rate | 0.59  (0.24-1.22) | 0.40  (0.05-1.44) | 0.44  (0.01-2.43) | 0.940 | 0.89  (0.24-2.28) | 0.352 |
| **Chronic kidney disease** | | | | | | |
| Events (n) | 263 | 123 | 78 | - | 62 | - |
| Incidence rate | 23.93  (21.13-27.01) | 26.20  (21.78-31.26) | 37.96  (30.00-47.37) | 0.010 | 14.62  (11.21-18.75) | <0.001 |
| **End-stage renal disease** | | | | | | |
| Events (n) | 13 | 6 | 5 | - | 2 | - |
| Incidence rate | 1.10  (0.59-1.88) | 1.19  (0.44-2.60) | 2.18  (0.71-5.10) | 0.318 | 0.44  (0.05-1.60) | 0.226 |
| **All-site cancer** | | | | | | |
| Events (n) | 67 | 27 | 20 | - | 20 | - |
| Incidence rate | 5.73  (4.44-7.28) | 5.43  (3.58-7.90) | 8.83  (5.39-13.64) | 0.099 | 4.49  (2.74-6.93) | 0.520 |
| **Hospitalization** | | | | | | |
| Events (n) | 643 | 301 | 157 | - | 185 | - |
| Incidence rate | 66.68  (61.62-72.04) | 73.51  (65.43-82.30) | 88.35  (75.07-103.30) | 0.062 | 49.05  (42.24-56.65) | <0.001 |
| 1. **JADE risk levels 3** | | | | | | |
| **Any major clinical events** | | | | | | |
| Events (n) | 4810 | 2894 | 1102 | - | 814 | - |
| Incidence rate | 105.29  (102.33-108.31) | 117.10  (112.87-121.44) | 129.22  (121.70-137.08) | 0.005 | 65.42  (61.01-70.08) | <0.001 |
| **All-cause death** | | | | | | |
| Events (n) | 778 | 468 | 180 | - | 130 | - |
| Incidence rate | 12.20  (11.36-13.09) | 13.25  (12.07-14.50) | 14.66  (12.60-16.97) | 0.247 | 8.05  (6.72-9.55) | <0.001 |
| **Coronary heart disease** | | | | | | |
| Events (n) | 461 | 285 | 82 | - | 94 | - |
| Incidence rate | 7.38  (6.73-8.09) | 8.25  (7.32-9.27) | 6.78  (5.39-8.42) | 0.117 | 5.95  (4.81-7.28) | 0.006 |
| **Myocardial infarction** | | | | | | |
| Events (n) | 205 | 127 | 46 | - | 32 | - |
| Incidence rate | 3.24  (2.81-3.71) | 3.62  (3.02-4.31) | 3.78  (2.76-5.04) | 0.809 | 1.99  (1.36-2.82) | 0.003 |
| **Peripheral vascular disease** | | | | | | |
| Events (n) | 109 | 58 | 36 | - | 15 | - |
| Incidence rate | 1.72  (1.41-2.07) | 1.65  (1.25-2.13) | 2.95  (2.07-4.08) | 0.006 | 0.93  (0.52-1.54) | 0.049 |
| **Stroke** | | | | | | |
| Events (n) | 319 | 171 | 76 | - | 72 | - |
| Incidence rate | 5.07  (4.53-5.66) | 4.90  (4.19-5.69) | 6.29  (4.96-7.88) | 0.070 | 4.53  (3.54-5.70) | 0.570 |
| **Heart failure** | | | | | | |
| Events (n) | 222 | 140 | 51 | - | 31 | - |
| Incidence rate | 3.51  (3.06-4.00) | 4.00  (3.36-4.72) | 4.19  (3.12-5.50) | 0.777 | 1.93  (1.31-2.74) | <0.001 |
| **Chronic kidney disease** | | | | | | |
| Events (n) | 4329 | 2614 | 1010 | - | 705 | - |
| Incidence rate | 91.70  (88.99-94.47) | 102.08  (98.21-106.07) | 115.19  (108.20-122.52) | 0.001 | 54.93  (50.95-59.13) | <0.001 |
| **End-stage renal disease** | | | | | | |
| Events (n) | 747 | 449 | 190 | - | 108 | - |
| Incidence rate | 12.02  (11.17-12.91) | 13.06  (11.88-14.33) | 16.01  (13.81-18.45) | 0.019 | 6.79  (5.57-8.20) | <0.001 |
| **All-site cancer** | | | | | | |
| Events (n) | 626 | 385 | 116 | - | 125 | - |
| Incidence rate | 10.02  (9.25-10.84) | 11.14  (10.05-12.31) | 9.61  (7.94-11.52) | 0.162 | 7.90  (6.58-9.41) | 0.001 |
| **Hospitalization** | | | | | | |
| Events (n) | 5384 | 3181 | 1271 | - | 932 | - |
| Incidence rate | 117.93  (114.80-121.13) | 127.39  (123.00-131.90) | 153.20  (144.89-161.86) | <0.001 | 75.24  (70.49-80.23) | <0.001 |
| 1. **JADE risk levels 4** | | | | | | |
| **Any major clinical events** | | | | | | |
| Events (n) | 2632 | 1644 | 600 | - | 388 | - |
| Incidence rate | 308.35  (296.68-320.36) | 321.53  (306.17-337.46) | 332.45  (306.37-360.14) | 0.484 | 239.81  (216.54-264.90) | <0.001 |
| **All-cause death** | | | | | | |
| Events (n) | 699 | 454 | 136 | - | 109 | - |
| Incidence rate | 37.40  (34.68-40.28) | 40.60  (36.95-44.52) | 34.88  (29.26-41.26) | 0.120 | 30.21  (24.80-36.44) | 0.006 |
| **Coronary heart disease** | | | | | | |
| Events (n) | 559 | 320 | 126 | - | 113 | - |
| Incidence rate | 32.76  (30.10-35.59) | 30.96  (27.66-34.55) | 35.16  (29.29-41.86) | 0.227 | 35.94  (29.62-143.21) | 0.173 |
| **Myocardial infarction** | | | | | | |
| Events (n) | 233 | 141 | 62 | - | 30 | - |
| Incidence rate | 12.81  (11.22-14.56) | 12.92  (10.88-15.24) | 16.37  (12.55-20.99) | 0.120 | 8.59  (5.80-12.27) | 0.043 |
| **Peripheral vascular disease** | | | | | | |
| Events (n) | 193 | 125 | 36 | - | 32 | - |
| Incidence rate | 10.60  (9.16-12.21) | 11.50  (9.57-13.70) | 9.43  (6.61-13.06) | 0.295 | 9.11  (6.23-12.86) | 0.239 |
| **Stroke** | | | | | | |
| Events (n) | 282 | 169 | 63 | - | 50 | - |
| Incidence rate | 15.63  (13.86-17.57) | 15.62  (13.35-18.16) | 16.71  (12.84-21.39) | 0.646 | 14.50  (10.77-19.12) | 0.646 |
| **Heart failure** | | | | | | |
| Events (n) | 412 | 264 | 88 | - | 60 | - |
| Incidence rate | 23.20  (21.01-25.55) | 24.94  (22.03-28.14) | 23.60  (18.93-29.07) | 0.652 | 17.40  (13.28-22.39) | 0.012 |
| **Chronic kidney disease** | | | | | | |
| Events (n) | 2367 | 1490 | 535 | - | 342 | - |
| Incidence rate | 249.84  (239.88-260.11) | 265.88  (252.55-279.73) | 264.75  (242.79-288.17) | 0.933 | 184.95  (165.87-205.63) | <0.001 |
| **End-stage renal disease** | | | | | | |
| Events (n) | 690 | 437 | 156 | - | 97 | - |
| Incidence rate | 39.91  (36.99-43.01) | 42.37  (38.49-46.54) | 43.71  (37.12-51.13) | 0.739 | 28.49  (23.10-34.76) | <0.001 |
| **All-site cancer** | | | | | | |
| Events (n) | 203 | 125 | 40 | - | 38 | - |
| Incidence rate | 11.06  (9.59-12.70) | 11.38  (9.47-13.56) | 10.41  (7.44-14.18) | 0.625 | 10.79  (7.64-14.82) | 0.776 |
| **Hospitalization** | | | | | | |
| Events (n) | 2571 | 1609 | 586 | - | 376 | - |
| Incidence rate | 257.14  (247.30-267.28) | 264.36  (251.60-277.60) | 287.67  (264.85-311.94) | 0.080 | 200.53  (180.77-221.86) | <0.001 |

Footnotes: Any major clinical event was defined as the first occurrence of either coronary heart disease, peripheral vascular disease, stroke, heart failure, chronic kidney disease, end-stage renal disease, all-site cancer, or all-cause death. Myocardial infarction was defined as either acute myocardial infarction or coronary artery bypass graft surgery. Hospitalization was calculated for patients with at least an overnight stay. We compared the incidence rates of clinical events and hospitalization using the Poisson regression model, with the JADE group as the referent. Definitions of JADE risk levels:

Level 1: No cardiovascular disease and end-stage renal disease; and having other condition fulfilling the “Low-risk” (one or less stratification parameters, and risk scores below the high sensitivity cut-off in all of the risk equations and eGFR≥90 ml/min/1.73m^2^).

Level 2: No cardiovascular disease and end-stage renal disease; none of the conditions (risk score, stratification parameters, eGFR) defined in the “High-risk” category but not belonging to the “Low-risk” category.

Level 3: No cardiovascular disease and end-stage renal disease and having three or more stratification parameters and/or risk scores above the high specificity cut-off in any one of the risk equations and/or eGFR<60 ml/min/1.73m^2^.

Level 4: Presence of any cardiovascular disease (coronary heart disease, heart failure, stroke, and/or peripheral vascular disease with or without interventions or medications) and/or end-stage renal disease (eGFR<15 ml/min/1.73m^2^ or need for renal replacement therapy).
