# Supplementary material for: Association of technologically assisted integrated care with clinical outcomes in type 2 diabetes in Hong Kong using the prospective JADE Program: A retrospective cohort analysis
Source: PLoS Med. 2020 Oct 2;17(10):e1003367. doi: 10.1371/journal.pmed.1003367 (PMC7531841; doi:10.1371/journal.pmed.1003367)
Supplement: S6 Table — (DOCX) [file pmed.1003367.s006.docx]

**S6 Table.** Cause-specific death rates (cases *per* 1000-patient-years) in all patients with type 2 diabetes and stratified by the non-JADE, JADE, and JADE-P groups (before propensity score-matching).

|  | **All patients** | **JADE**  **(publicly-funded evaluation, JADE report, and group education; referent group)** | **Non-JADE**  **(publicly-funded**  **evaluation)** | ***P*-value**  **(non-JADE *vs.* JADE)** | **JADE-P**  **(self-paid evaluation,**  **JADE report, personalized empowerment, and annual telephone reminder for engagement)** | ***P*-value (JADE-P *vs.* JADE)** |
| --- | --- | --- | --- | --- | --- | --- |
| **Death from all-causes** | | | | | | |
| Events (n) | 1523 | 947 | 329 | - | 247 | - |
| Incidence rate | 16.15  (15.36-16.98) | 18.36  (17.21-19.57) | 17.81  (15.94-19.84) | 0.631 | 10.18  (8.95-11.53) | <0.001 |
| **Vascular deaths** | | | | | | |
| Events (n) | 283 | 185 | 50 | - | 48 | - |
| Incidence rate | 3.00  (2.66-3.37) | 3.59  (3.09-4.14) | 2.71  (2.01-3.57) | 0.077 | 1.98  (1.46-2.62) | <0.001 |
| **Cancer deaths** | | | | | | |
| Events (n) | 302 | 192 | 54 | - | 56 | - |
| Incidence rate | 3.20  (2.85-3.58) | 3.72  (3.22-4.29) | 2.92  (2.20-3.81) | 0.116 | 2.31  (1.74-3.00) | 0.002 |
| **Non-vascular, non-cancer deaths** | | | | | | |
| Events (n) | 665 | 416 | 156 | - | 93 | - |
| Incidence rate | 7.05  (6.53-7.61) | 8.07  (7.31-8.88) | 8.44  (7.17-9.88) | 0.627 | 3.83  (3.09-4.69) | <0.001 |
| **Deaths due to ill-defined causes** | | | | | | |
| Events (n) | 273 | 154 | 69 | - | 50 | - |
| Incidence rate | 2.89  (2.56-3.26) | 2.99  (2.53-3.50) | 3.73  (2.91-4.73) | 0.123 | 2.06  (1.53-2.72) | 0.023 |

Footnotes: The classification of cause-specific death was based on the National Health and Interview Survey (NHIS 1986-2004) Linked Mortality Files (<https://www.cdc.gov/nchs/data/datalinkage/underlying_and_multiple_causes_of_death557.pdf>. Accessed on 3 May 2018). We compared the incidence rates of cause-specific deaths using the Poisson regression model, with the JADE group as the referent.
