# Supplementary material for: Association of technologically assisted integrated care with clinical outcomes in type 2 diabetes in Hong Kong using the prospective JADE Program: A retrospective cohort analysis
Source: PLoS Med. 2020 Oct 2;17(10):e1003367. doi: 10.1371/journal.pmed.1003367 (PMC7531841; doi:10.1371/journal.pmed.1003367)
Supplement: S7 Table — (DOCX) [file pmed.1003367.s007.docx]

**S7 Table.** Baseline characteristics of patients with type 2 diabetes, stratified by the status of loss to follow-up (yes/no) defined as lack of data in the public healthcare system one year before censoring or death, and before propensity score-matching.

|  | **Yes** | **No** |
| --- | --- | --- |
| Number | 1257 | 15,366 |
| Observation period^*^ (years) | 6.4 (4.6-7.8) | 5.9 (4.2-7.0) |
| Age (years) | 55.9±12.2 | 60.6±11.4 |
| Diabetes duration^*^ (years) | 4.0 (1.0-9.0) | 8.0 (2.0-14.0) |
| Age of diagnosis (years) | 51.3±11.0 | 52.1±11.1 |
| Men, n (%) | 804 (64.0%) | 8266 (53.8%) |
| Former/current smoker, n (%) | 387 (30.8%) | 4947 (32.2%) |
| At least college education, n (%) | 312 (32.6%) | 1576 (18.2%) |
| **JADE risk level, n (%)** | | |
| 1-2^†^ | 252 (20.0%) | 1685 (11.0%) |
| 3 | 836 (66.5%) | 10,178 (66.2%) |
| 4 | 169 (13.4%) | 3501 (22.8%) |
| **Past medical history, n (%)** | | |
| Coronary heart disease | 119 (9.5%) | 2026 (13.2%) |
| Peripheral vascular disease | 21 (1.7%) | 620 (4.0%) |
| Stroke | 54 (4.3%) | 1369 (8.9%) |
| Heart failure | 17 (1.4%) | 596 (3.9%) |
| Chronic kidney disease | 98 (7.8%) | 3114 (20.3%) |
| End-stage renal disease | 4 (0.3%) | 261 (1.7%) |
| Sensory neuropathy | 47 (3.7%) | 919 (6.0%) |
| Diabetic retinopathy | 240 (19.2%) | 4242 (27.6%) |
| All-site cancer | 45 (3.6%) | 948 (6.2%) |
| **Clinical assessment** | | |
| Waist circumference (men; cm) | 91.8±10.1 | 91.9±10.8 |
| Waist circumference (women; cm) | 86.0±10.4 | 87.3±11.2 |
| Systolic blood pressure (mmHg) | 131.0±17.3 | 136.0±18.9 |
| Diastolic blood pressure (mmHg) | 78.3±10.1 | 78.2±10.7 |
| **Biochemical assessment** | | |
| HbA_1c_ (%) | 7.37±1.59 | 7.61±1.58 |
| HbA_1c_ (mmol/mol) | 57.0±17.4 | 60.0±17.3 |
| Triglyceride^*^ (mmol/L) | 1.3 (0.9-1.9) | 1.3 (0.9-1.9) |
| HDL-cholesterol (mmol/L) | 1.29±0.35 | 1.31±0.37 |
| LDL-cholesterol (mmol/L) | 2.60±0.87 | 2.59±0.89 |
| Urinary albumin:creatinine ratio^*^ (mg/mmol) | 1.0 (0.5-3.6) | 2.0 (0.7-9.6) |
| Estimated glomerular filtration rate (ml/min/1.73m^2^) | 88.1±19.6 | 79.5±24.0 |
| **Treatment targets attainment at baseline, n (%)** | | |
| HbA_1c_<7% (53 mmol/mol) | 659 (52.5%) | 6238 (40.7%) |
| Blood pressure<130/80 mmHg | 530 (42.2%) | 5048 (32.9%) |
| LDL-cholesterol<2.6 mmol/L | 619 (50.4%) | 7919 (52.5%) |
| At least 2 treatment targets attained | 596 (47.4%) | 5741 (37.4%) |
| **Medication usage at baseline, n (%)** | | |
| Insulin | 122 (9.7%) | 4030 (26.2%) |
| Oral glucose-lowering agents | 979 (77.9%) | 13,056 (85.0%) |
| RASi | 460 (36.6%) | 7508 (48.9%) |
| Statins | 466 (37.1%) | 6977 (45.4%) |
| **Self-care activity in last three months, n (%)** | | |
| SMBG at least weekly | 563 (48.7%) | 7421 (52.2%) |
| Physical activity at least three times/week | 537 (42.8%) | 7252 (47.4%) |
| Adherence to balanced diet | 1096 (87.3%) | 13,807 (90.1%) |
| At least 2 self-care activities | 771 (61.3%) | 10,276 (66.9%) |
| **Distribution in groups, n (%)** | | |
| Non-JADE | 114 (9.1%) | 3471 (22.6%) |
| JADE | 352 (28.0%) | 9253 (60.2%) |
| JADE-P | 791 (62.9%) | 2642 (17.2%) |

Footnotes: Data are expressed in mean±standard deviation, median (interquartile range)^*^, and number (percentages), as appropriate. ^†^We combined JADE risk levels 1 and 2 due to small sample size. HDL-cholesterol, high-density lipoprotein cholesterol; LDL-cholesterol, low-density lipoprotein cholesterol; NA, not applicable; RAS, renin-angiotensin system; SMBG, self-monitoring of blood glucose. SI conversion factors: To convert LDL-cholesterol and HDL-cholesterol to mg/dL, multiply by 38.67. To convert triglyceride to mg/dL, multiply by 88.57.
