# Supplementary material for: Association of technologically assisted integrated care with clinical outcomes in type 2 diabetes in Hong Kong using the prospective JADE Program: A retrospective cohort analysis
Source: PLoS Med. 2020 Oct 2;17(10):e1003367. doi: 10.1371/journal.pmed.1003367 (PMC7531841; doi:10.1371/journal.pmed.1003367)
Supplement: S8 Table — (DOCX) [file pmed.1003367.s008.docx]

**S8 Table.** The levels of key risk factors at baseline and study end in the non-JADE and JADE groups (after propensity score-matching).

|  | **Non-JADE** | | **JADE** | |
| --- | --- | --- | --- | --- |
|  | **Baseline** | **Study end** | **Baseline** | **Study end** |
| Duration of follow-up^*^ | -- | 5.7 (4.0-6.7) | -- | 5.9 (4.2-6.8) |
| HbA_1c_ (%) | 7.75±1.57 | 7.61±1.41 | 7.75±1.69 | 7.44±1.30 |
| HbA_1c_ (mmol/mol) | 61.0±17.2 | 60.0±15.4 | 61.0±18.5 | 58.0±14.2 |
| Triglyceride^*^ (mmol/L) | 1.4 (1.0-1.9) | 1.3 (1.0-1.9) | 1.3 (0.9-1.9) | 1.3 (0.9-1.9) |
| LDL-cholesterol (mmol/L) | 2.78±0.93 | 2.26±0.74 | 2.77±0.90 | 2.18±0.75 |
| Urinary albumin:creatinine ratio^*^ (mg/mmol) | 2.1 (0.7-9.1) | 2.4 (0.8-14.1) | 1.7 (0.6-7.9) | 2.0 (0.7-10.6) |
| Estimated glomerular filtration rate (ml/min/1.73m^2^) | 81.2±23.3 | 69.5±27.8 | 81.1±22.4 | 71.8±27.9 |

Footnotes: Data are expressed in mean±standard deviation and median (interquartile range)^*^, as appropriate. Data at study end were based on the last available laboratory measurements captured in the territory-wide Electronic Medical Record system. To convert LDL-cholesterol to mg/dL, multiply by 38.67. To convert triglyceride to mg/dL, multiply by 88.57.
