# Supplementary material for: Association of technologically assisted integrated care with clinical outcomes in type 2 diabetes in Hong Kong using the prospective JADE Program: A retrospective cohort analysis
Source: PLoS Med. 2020 Oct 2;17(10):e1003367. doi: 10.1371/journal.pmed.1003367 (PMC7531841; doi:10.1371/journal.pmed.1003367)
Supplement: S9 Table — (DOCX) [file pmed.1003367.s009.docx]

**S9 Table.** The levels of key risk factors at baseline and study end in the JADE-P and JADE groups (after propensity score-matching).

|  | **JADE-P** | | **JADE** | |
| --- | --- | --- | --- | --- |
|  | **Baseline** | **Study end** | **Baseline** | **Study end** |
| Duration of follow-up^*^ | -- | 7.9 (5.7-8.8) | -- | 5.8 (4.2-6.8) |
| HbA_1c_ (%) | 7.36±1.59 | 7.24±1.23 | 7.37±1.43 | 7.32±1.26 |
| HbA_1c_ (mmol/mol) | 57.0±17.4 | 56.0±13.4 | 57.0±15.6 | 56.0±13.8 |
| Triglyceride^*^ (mmol/L) | 1.4 (1.0-1.9) | 1.2 (0.9-1.7) | 1.3 (0.9-1.9) | 1.3 (0.9-1.9) |
| LDL-cholesterol (mmol/L) | 2.66±0.94 | 2.18±0.75 | 2.63±0.83 | 2.13±0.71 |
| Urinary albumin:creatinine ratio^*^ (mg/mmol) | 1.2 (0.5-4.4) | 1.4 (0.6-5.7) | 1.4 (0.5-5.3) | 1.7 (0.6-7.2) |
| Estimated glomerular filtration rate (ml/min/1.73m^2^) | 82.3±20.3 | 74.4±26.0 | 82.1±21 | 74.4±25.1 |

Footnotes: Data are expressed in mean±standard deviation and median (interquartile range)^*^, as appropriate. Data at study end were based on the last available laboratory measurements captured in the territory-wide Electronic Medical Record system. To convert LDL-cholesterol to mg/dL, multiply by 38.67. To convert triglyceride to mg/dL, multiply by 88.57.
