# Supplementary figures and images for: Association of technologically assisted integrated care with clinical outcomes in type 2 diabetes in Hong Kong using the prospective JADE Program: A retrospective cohort analysis
Source: PLoS Med. 2020 Oct 2;17(10):e1003367. doi: 10.1371/journal.pmed.1003367 (PMC7531841; doi:10.1371/journal.pmed.1003367)

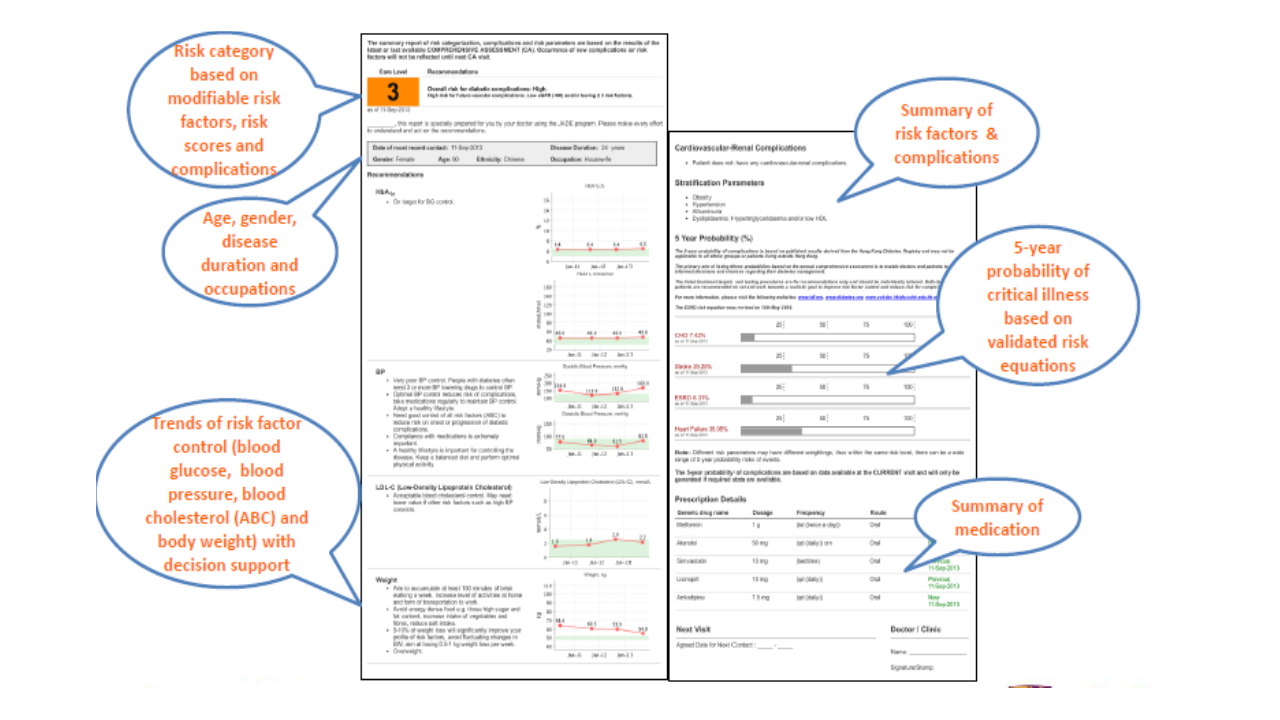

Supplement: S1 Fig — (TIF) [file pmed.1003367.s010.tif]

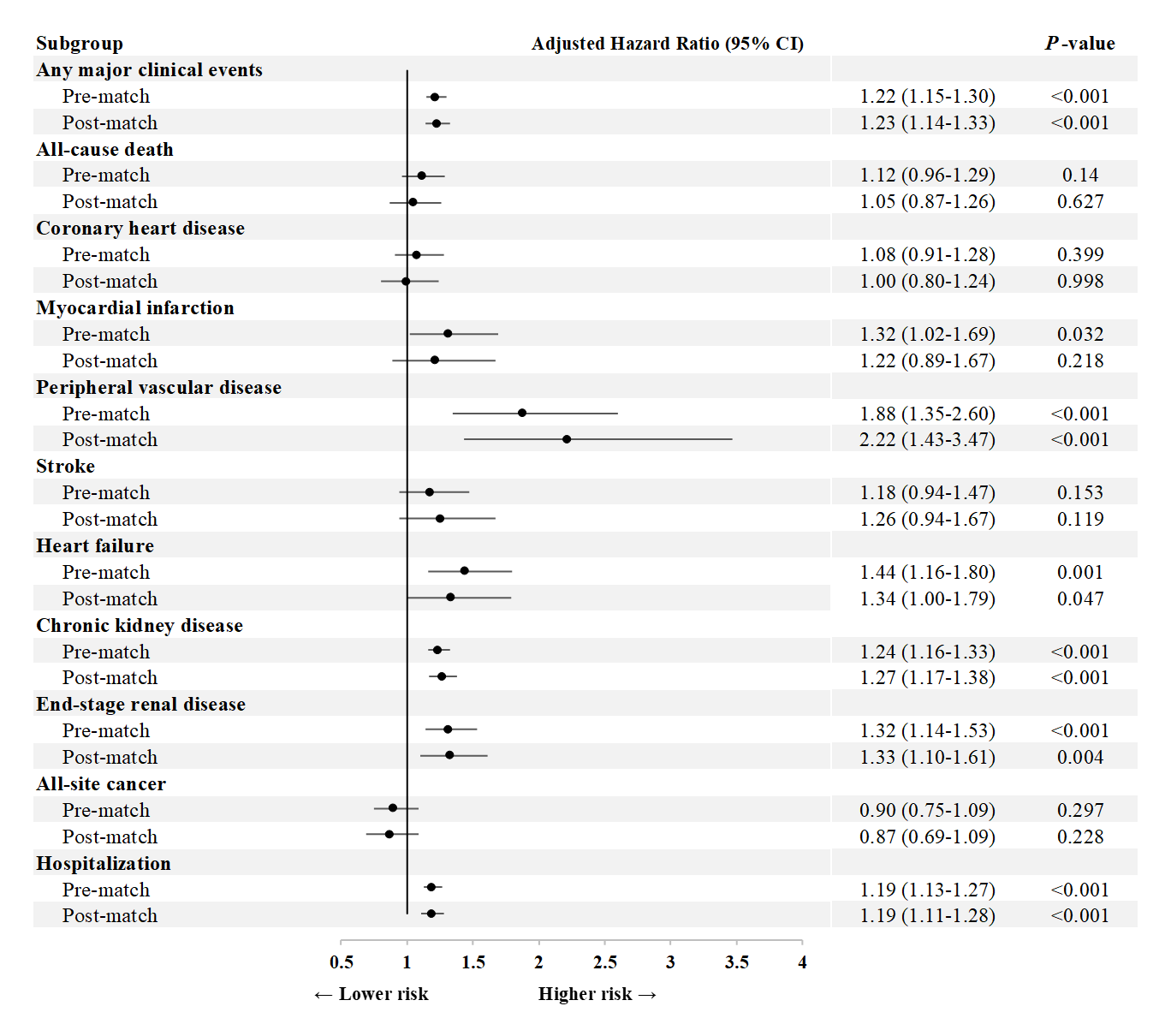

Supplement: S2 Fig — (TIF) [file pmed.1003367.s011.tif]

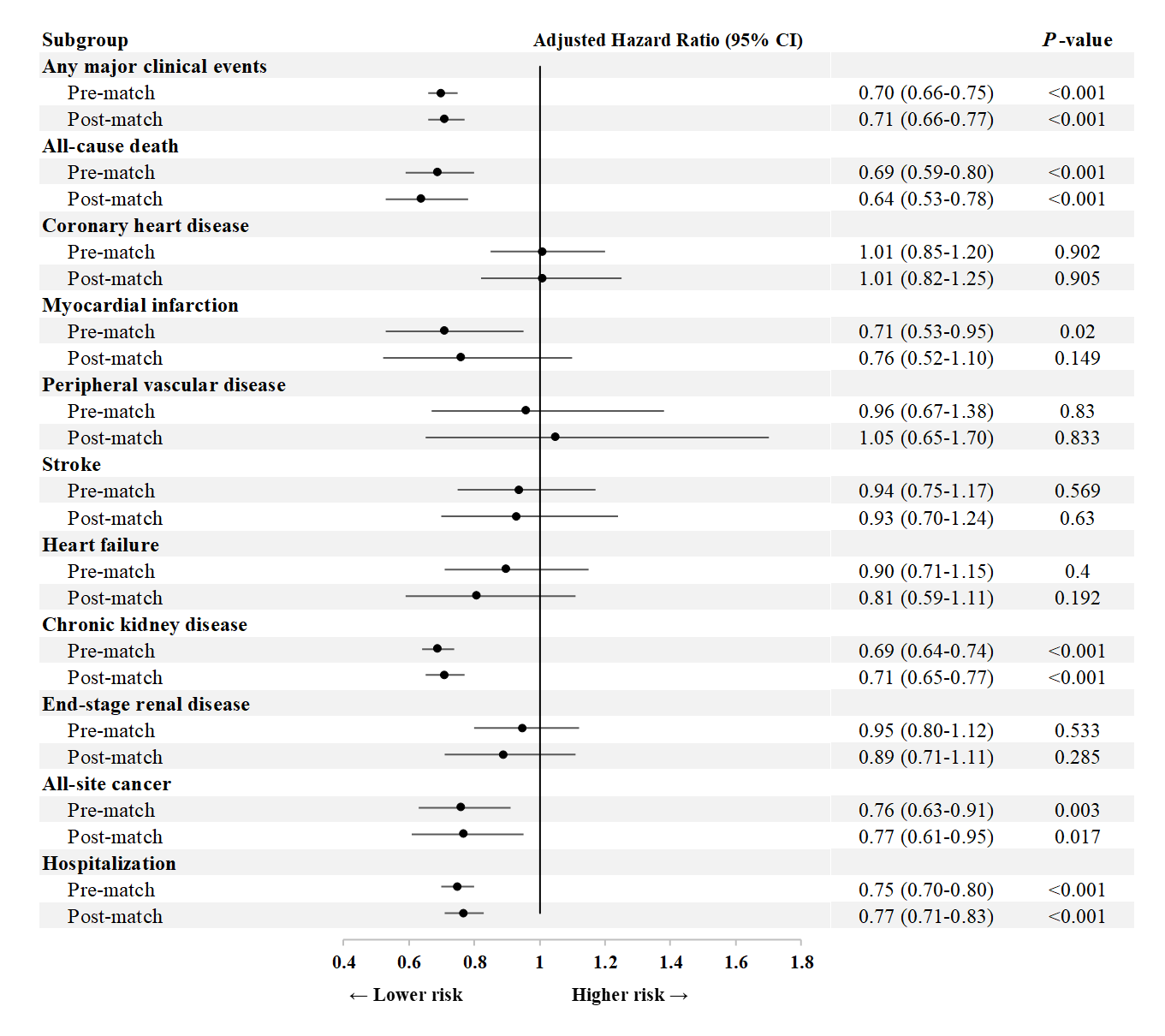

Supplement: S3 Fig — (TIF) [file pmed.1003367.s012.tif]

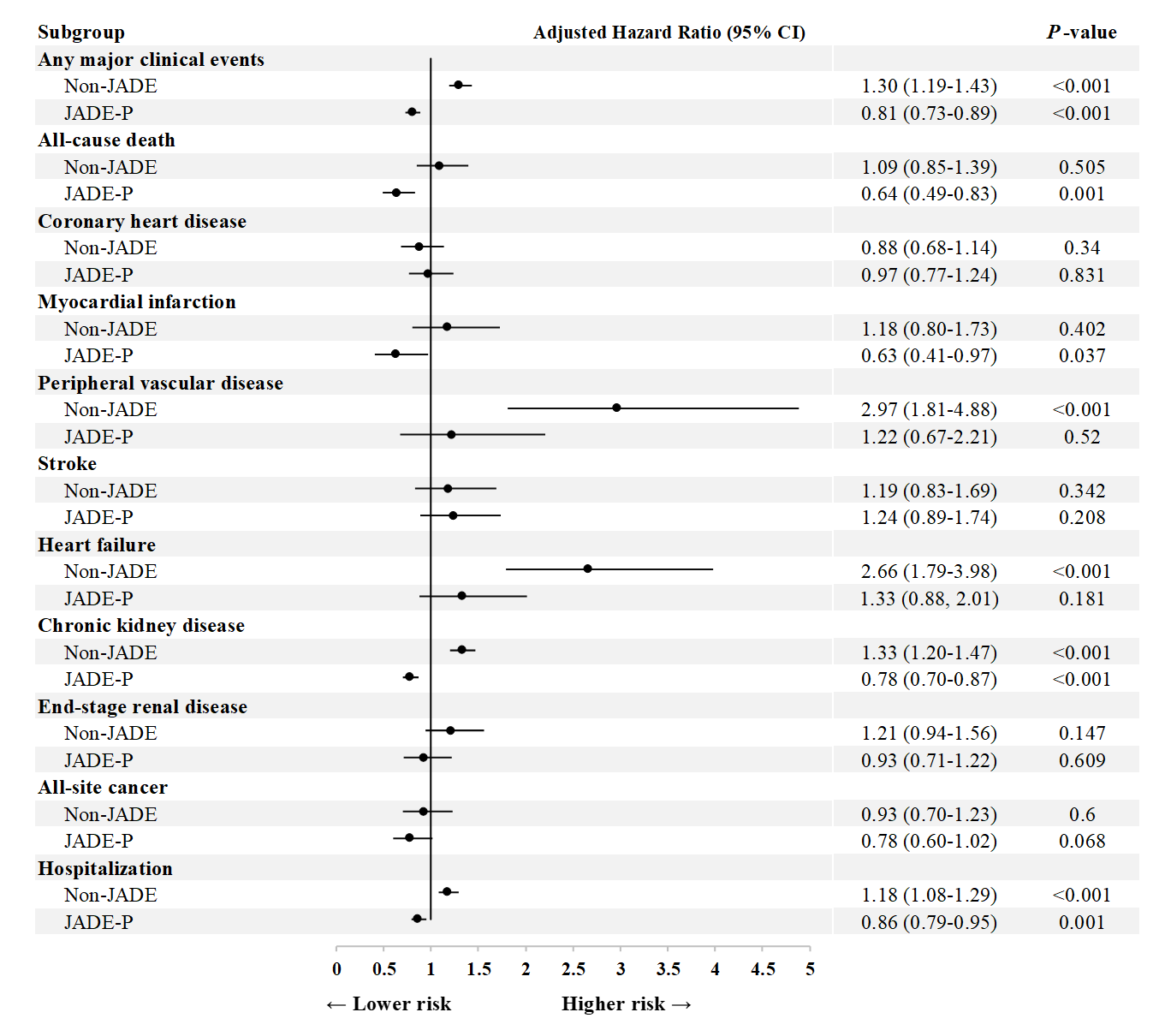

Supplement: S4 Fig — (TIF) [file pmed.1003367.s013.tif]
