# Supplementary material for: Association of technologically assisted integrated care with clinical outcomes in type 2 diabetes in Hong Kong using the prospective JADE Program: A retrospective cohort analysis
Source: PLoS Med. 2020 Oct 2;17(10):e1003367. doi: 10.1371/journal.pmed.1003367 (PMC7531841; doi:10.1371/journal.pmed.1003367)
Supplement: S1 Text — (DOCX) [file pmed.1003367.s014.docx]

**S1 Text.** Economic evaluation of the present JADE Program

The main implementation cost is nursing labour cost and the licensing/development fee of the JADE portal. In Hong Kong, the average hourly salary for a registered nurse is estimated at US$ 30. The average cost per inpatient day is estimated at US$ 660.

**Nurse hours per year in three care settings**

|  | **JADE** | **JADE-P** | **Non-JADE** |
| --- | --- | --- | --- |
| Nurse to patient ratio | 1:35 | 1:1 | -- |
| Total time spent during empowerment session (hour) | 2.5 | 0.5 | -- |
| Frequency of empowerment session per year | 0.5 | 1.0 | -- |
| Time spent per patient per year (hour) | (2.5^*^0.5) / 35 = 0.036 | (0.5^*^1.0) / 1 = 0.5 | -- |

The non-JADE group did not receive regular empowerment (refer Fig 2 in the main text for differences in the evaluation workflow in three care settings). JADE, Joint Asia Diabetes Evaluation; JADE-P, Joint Asia Diabetes Evaluation-Personalized.

**Estimated differences in costs per patient per year in three care settings (quoted in US$)**

|  | **Average inpatient days** | **Estimated hospitalization costs** | **Nurse hours** | **Estimated nursing costs per hour** | **Total estimated costs** | **Total costs difference** |
| --- | --- | --- | --- | --- | --- | --- |
| **JADE** | 3.2 | 2123 | 0.036 | 1.1 | 2124 | Reference |
| **JADE-P** | 1.9 | 1228 | 0.50 | 15.0 | 1243 | -880 |
| **Non-JADE** | 3.6 | 2377 | -- | -- | 2377 | 254 |

Costs are rounded off to the nearest integer. Currency exchange rate: US$ 1 = HK$ 7.75 (as of 31 May 2020).

**Reference**

1. Hospital Authority Public Charges – Non-eligible Persons. G.N. 2107 of 2020 (amendment effective from 29 January 2020). Available from <https://www.ha.org.hk/haho/ho/cs/238767_en.pdf>.
